# Magnetic Sponge with Neutral–Ionic Phase Transitions

**DOI:** 10.1002/advs.201700526

**Published:** 2017-12-04

**Authors:** Wataru Kosaka, Yusuke Takahashi, Masaki Nishio, Keisuke Narushima, Hiroki Fukunaga, Hitoshi Miyasaka

**Affiliations:** ^1^ Institute for Materials Research Tohoku University 2‐1‐1 Katahira, Aoba‐ku Sendai 980‐8577 Japan; ^2^ Department of Chemistry Graduate School of Science Tohoku University 6‐3 Aramaki, Aza‐Aoba, Aoba‐ku Sendai 980‐8578 Japan; ^3^ Department of Chemistry Division of Material Sciences Graduate School of Natural Science and Technology Kanazawa University Kakuma‐machi Kanazawa 920‐1192 Japan

**Keywords:** chain structures, donor–acceptor systems, host–guest chemistry, magnetic properties, neutral–ionic phase transitions

## Abstract

Phase transitions caused by the charge instability between the neutral and ionic phases of compounds, i.e., N–I phase transitions, provide avenues for switching the intrinsic properties of compounds related to electron/spin correlation and dipole generation as well as charge distribution. However, it is extremely difficult to control the transition temperature (*T*_c_) for the N–I phase transition, and only chemical modification based on the original material have been investigated. Here, a design overview of the tuning of N–I phase transition by interstitial guest molecules is presented. This study reports a new chain coordination‐polymer [Ru_2_(3,4‐Cl_2_PhCO_2_)_4_TCNQ(EtO)_2_]∙DCE (**1‐DCE**; 3,4‐Cl_2_PhCO_2_^−^ = 3,4‐dichlorobenzoate; TCNQ(EtO)_2_ 2,5‐diethoxy‐7,7,8,8‐tetracyanoquinodimethane; and DCE = 1,2‐dichloroethane) that exhibits a one‐step N–I transition at 230 K (= *T*_c_) with the N‐ and I‐states possessing a simple paramagnetic state and a ferrimagnetically correlated state for the high‐ and low‐temperature phases, respectively. The *T*_c_ continuously decreases depending on the content of DCE, which eventually disappears with the complete evacuation of DCE, affording solvent‐free compound **1** with the N‐state in the entire temperature range (this behavior is reversible). This is an example of tuning the in situ *T*_c_ for the N–I phase transition via the control of the interstitial guest molecules.

## 1 Introduction

Phase transitions involving electron transfer constitute one of the most intriguing phenomena that permit the modulation of the intrinsic physical properties of materials. The electronic states of materials and their spin states with superexchange interactions, transient electronic transport, electric polarization, and structural changes are tuned as a function of external stimuli. A representative example involves the phase transition between the neutral (N:D^0^A^0^) and ionic (I:D^+^A^−^) charge states (i.e., N–I phase transition). This example represents a new research field in organic electron‐donor (D)–acceptor (A) systems,^1^, ^2^, ^3^, ^4^, ^5^, ^6^, ^7^, ^8^ with the discovery of the first compound tetrathiafulvalene–chloranil in the class of alternating π‐stacking DA systems by Torrance et al.^9^, ^10^ However, these organic DA systems were of low interest from the viewpoint of spin systems because of the sole production of *S* = 1/2 antiferromagnetic pairs in the I‐state involving the structural distortion (i.e., dimerization). Conversely, a covalently bonded DA system exhibiting the N–I phase transition has been observed for a metal complex chain with the D and A of [Ru_2_(2,3,5,6‐F_4_PhCO_2_)_4_] and DMDCNQI, respectively, [Ru_2_(2,3,5,6‐F_4_PhCO_2_)_4_DMDCNQI]·2(*p*‐xylene) (**0**, 2,3,5,6‐F_4_PhCO_2_^−^ = 2,3,5,6‐tetrafluorobenzoate, DMDCNQI = 2,5‐dimethyl‐*N*,*N*′‐dicyanoquinonediimine).^11^ Ferrimagnetic correlation between the spins of *S* = 3/2 for [Ru_2_^II,III^]^+^ and *S* = 1/2 for DMDCNQI^•−^ in the I‐phase is produced via an electron transfer from the paramagnetic N‐phase with *S* = 1 of [Ru_2_^II,II^] without dimerization. Interestingly, this compound exhibited a stepwise transition via an intermediate state composed of N and I chains in a 1:1 ratio (average state of D^0.5+^A^0.5−^), which was caused by the effect of anisotropic interchain Coulomb interactions. This result implied that the electron transfer from D to A (or from A^−^ to D^+^) is also possibly sensitive to the environment in which D and A are located, because this could be associated with the Madelung stabilization in the I‐phase, and not just sensitive to the balance of intrinsic potentials for D/A, i.e., the ionization potential (*I*_D_) of D and the electron affinity (*E*_A_) of A. Indeed, the transition temperature (*T*_c_) is sensitive to the application of hydrostatic pressure and partial chemical modification in the same system.^11^, ^12^, ^13^ These results have motivated the research of chemically switchable N–I phase transitions; as the previous compound represented the only case wherein chains with multiple spin states exhibit the N–I phase transition, new systems have been considered for developing guest‐induced magnetic change (i.e., magnetic sponge) phenomena associated with the N–I phase transition.

This study reports an N–I phase transition compound in which the N–I phase transition behavior varies depending on its solvation. This compound is a 1D coordination polymer, [Ru_2_(3,4‐Cl_2_PhCO_2_)_4_TCNQ(EtO)_2_]·(DCE) (**1‐DCE**; 3,4‐Cl_2_PhCO_2_^−^ = 3,4‐dichlorobenzoate; TCNQ(EtO)_2_ = 2,5‐diethoxy‐7,7,8,8‐tetracyanoquinodimethane; DCE = 1,2‐dichloroethane), in which D and A moieties of [Ru_2_(3,4‐Cl_2_PhCO_2_)_4_] and TCNQ(EtO)_2_, respectively, undergo the one‐step temperature‐induced N–I phase transition at 230 K. Compound **1‐DCE** contains an interstitial solvent (DCE) between chains, which can be removed while maintaining its crystallinity, thereby affording its desolvated form, [Ru_2_(3,4‐Cl_2_PhCO_2_)_4_TCNQ(EtO)_2_] (**1**), without collapsing the chain form. Interestingly, **1** does not exhibit the N–I phase transition and maintains the N‐phase in the entire temperature range measured. The DCE solvation/desolvation (SD) transformation between **1** and **1‐DCE** is reversible. The *T*_c_ of the partially solvated compounds shifts depending on the degree of solvation of DCE. This study demonstrates that the N–I phase transition can be exploited for the continuous regulation of physical properties via the sensitive tuning of interstitial guest molecules.

## 2 Results and Discussion

### 2.1 Selection of D and A Units

The paddlewheel‐type diruthenium(II, II) complex, [Ru_2_^II,II^(3,4‐Cl_2_PhCO_2_)_4_], and TCNQ(EtO)_2_, were selected as the D and A units for searching a N–I phase transition compound. These DA systems exhibit the following remarkable features: (1) the *I*_D_ of [Ru_2_] and *E*_A_ of the TCNQ or DCNQI derivatives can be easily tuned by modifying the substitution of ligands or molecules without changing the basic assembly form; (2) the spin set in the I‐phase is not canceled even for an antiferromagnetically coupled set although it is canceled in the organic DA systems, leading to a ferrimagnetic spin correlation of *S* = 3/2 for [Ru_2_^II,III^]^+^ and *S* = 1/2 for TCNQ^•−^ through a chain; (3) the D^+^A^−^ set in the I‐state did not permit the dimerization that is frequently observed in π‐stacked organic systems, as a Peierls distortion^2^, ^3^ because of its robust covalently bonded framework. To predict the balance between the *I*_D_ of D and *E*_A_ of A, the (Δ*E*_H–L_(DA)) gap between the highest occupied molecular orbital level of D and lowest unoccupied molecular orbital level of A was easily evaluated by density functional theory calculations for each unit. A plot of Δ*E*_H–L_(DA) as a function of Δ*E*_1/2_(DA), wherein Δ*E*_1/2_(DA) is the potential gap between the redox potentials of D and A, well predicted the oxidation state of these DA and D_2_A assembly compounds (Figure S1, Supporting Information). The N‐ and I‐states are expected for Δ*E*_H–L_(DA) > 0 and Δ*E*_H–L_(DA) < 0, respectively.^14^, ^15^ Interestingly, the Δ*E*_H–L_(DA) value for the present DA set is 0.2291 eV.^16^, ^17^ This value is close to Δ*E*_H–L_(DA) = 0, i.e., the boundary region between N‐ and I‐states, which is similar to that (0.2003 eV) for the DA set of the previous N–I transition compound **0** (Figure S1, Supporting Information).^14^

Notably, the chain‐type compounds of the [Ru_2_^II,II^] units with bidentate linkers often exhibit a porous nature for interstitial crystallization solvents or guest gas molecules.^18^, ^19^, ^20^, ^21^, ^22^ This characteristic led us to prepare guest‐induced switching materials associated with the N–I phase transition, as reported for some porous coordination polymers (PCPs) in terms of spin states,^23^, ^24^, ^25^ electrical conductivity,^26^, ^27^ and magnetic ordering.^28^, ^29^, ^30^, ^31^, ^32^, ^33^, ^34^, ^35^

### 2.2 Variation in the Structure of **1‐DCE** According to the N–I Phase Transition

The **1‐DCE** structure was examined by single crystal X‐ray crystallography at several temperatures ranging from 103 to 270 K. First, a change in the lattice system suitable for the international union of crystallography (IUCr) rule was observed with a change in the temperature;^36^ however, the Miller indices based on the cell system at 103 K were typically used for conveniently referring the crystallographic directions and planes for all data in the following discussions.

**Figure 1:**
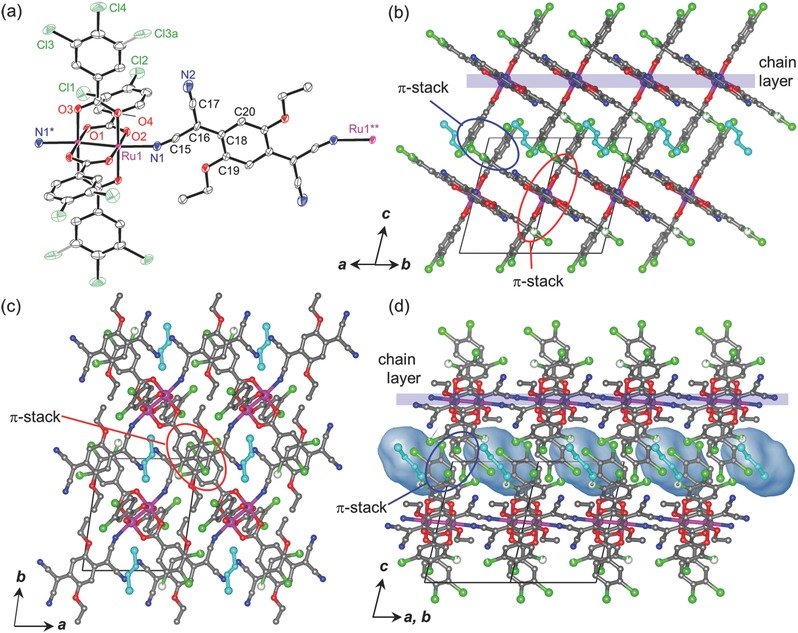
Crystal structure of **1‐DCE** at 103 K. a) Thermal ellipsoid plot showing the formula unit and atom numbering scheme. Displacement ellipsoids are drawn at a 50% probability level. Gray bond indicates the minor component of the positional disorder. Packing diagrams projected along the b) <110> direction (chain‐running direction), c) *c* axis (top view of the chain layer), and d) <1−10> direction (side view of the chain layer). Blue, red, gray, green, and purple represent N, O, C, F, and Ru, respectively. Hydrogen atoms and crystal solvents in (a) are omitted for clarity. Crystal solvents in (b)–(d) are colored in cyan. Connolly surfaces are also depicted in (d).

Compound **1‐DCE** crystallized in the triclinic *P*‐1 space group in the entire temperature range was investigated (Tables S1–S5, Supporting Information), wherein one half of the formula unit, with inversion centers at the midpoint of the Ru—Ru bond and the center of the TCNQ(EtO)_2_ and DCE molecule, was determined as an asymmetric unit (*Z* = 1). **Figure** **1**a depicts the ORTEP drawing of the formula unit of **1‐DCE** at 103 K with atomic numbering schemes; chlorine atoms at the *meta* positions of the benzoate moiety exhibited disordered positions. Table S2 (Supporting Information) summarizes the selected bond distances and angles. Although TCNQ(EtO)_2_ can serve as a tetradentate ligand with four cyano groups, the moiety in **1‐DCE** acted as a linear‐type bidentate ligand with only two cyano groups in the *trans* position, affording a DA alternating chain motif with an [—(Ru_2_)—{TCNQ(EtO)_2_}—] repeat similar to **0**,^11^ and an ionic DA chain compound with bis(1,2,5‐thiadiazolo)tetracyanoquinodimethane (BTDA‐TCNQ).^37^ The chains run along the <110> direction, affording a chain‐aggregated layer on the (001) plane (hereafter known as a chain layer; see Figure 1b–d). Within the chain layer, chains were closely packed in an antiphase manner (i.e., ⋅⋅⋅D⋅⋅⋅A⋅⋅⋅D⋅⋅⋅A⋅⋅⋅) along the <1−10> direction, with the interchain π‐stack stabilization related to each of the TCNQ molecules sandwiched from the top and bottom by the phenyl ring of the benzoate moieties in the neighboring chains (red circles in Figure 1), leading to a relatively short interchain [Ru_2_]⋅⋅⋅TCNQ (centroid to centroid) distance of 7.39 Å at 103 K (the <1−10> direction, Table S5, Supporting Information). One DCE molecule was present as an interstitial crystallization solvent between the chain layers, occupying an isolated pore with a solvent‐accessible volume of 118 Å^3^ (9%) at 103 K (Figure 1d shows the Connolly surfaces). Thus, the chains along the <001> direction (i.e., between chain layers) are significantly separated with 12.10 Å at 103 K (Table S5, Supporting Information), although π–π stacks are formed between benzoate groups because of the in‐phase alignment (i.e., ⋅⋅⋅D⋅⋅⋅D⋅⋅⋅D⋅⋅⋅ or ⋅⋅⋅A⋅⋅⋅A⋅⋅⋅A⋅⋅⋅) along the <001> direction (blue circles in Figure 1).

**Figure 2:**
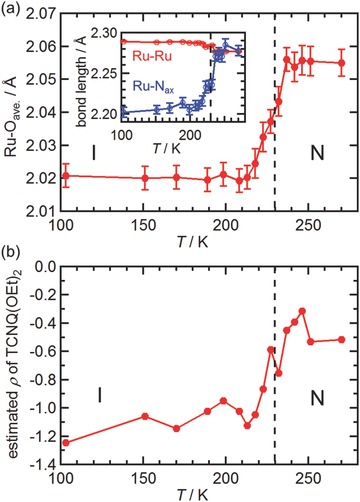
Variation in the structure of **1‐DCE** as a function of temperature. a) Variation in the average Ru—O_eq_ bond (O_eq_ = carboxylate oxygen atoms), where the inset shows the variations in the Ru—Ru and Ru—N_ax_ bonds (N_ax_ = cyano nitrogen atom of TCNQ(EtO)_2_). b) The degree of charge ρ on the TCNQ(EtO)_2_ subunit estimated by the Kistenmacher relationship.

The Ru—O_eq_ length (O_eq_ = equatorial oxygen atoms) was extremely sensitive to the oxidation state of the [Ru_2_] unit. Typically, this length has been reported to be 2.06–2.07 Å for [Ru_2_^II,II^] and 2.02–2.03 Å for [Ru_2_^II,III^]^+^.^38^ **Figure** **2**a plots the average Ru—O_eq_ bond length (Table S3, Supporting Information) as a function of temperature. At 270 K, the average Ru—O_eq_ bond length was 2.056(4) Å, indicative of the [Ru_2_^II,II^] oxidation state. As a result of cooling, the average bond length decreased to around 2.02 Å in the temperature range of 237–213 K. At temperatures less than 213 K, the Ru—O_eq_ bond length was maintained at ≈2.02 Å for [Ru_2_^II,III^]^+^. In addition, the axial bond length (Ru—N_ax_), which was also sensitive to the oxidation state of the [Ru_2_] unit,^11^ suddenly became shorter on cooling at temperatures less than 237 K (Table S3 (Supporting Information) and inset of Figure 2a). In addition, the Ru—Ru bond length exhibited a small anomaly around 230 K (Table S3 (Supporting Information) and inset of Figure 2a).^11^

The bond lengths in the TCNQ(EtO)_2_ moieties reflected the oxidation state (ρ) of the TCNQ(EtO)_2_ moieties (Table S4, Supporting Information), which can be typically evaluated using the Kistenmacher relationship: ρ = −{*A*[*c*/(*b* + *d*)] + *B*},^39^ where *b*, *c*, and *d* are the C—C bond distances between 7,9‐, 1,7‐, and 1,2‐positions in the TCNQ(EtO)_2_ moiety, respectively, and the parameters *A* = −41.667 and *B* = 19.833 are determined by assuming a completely neutral form of TCNQ^0^ (ρ = 0)^40^ and a one‐electron‐reduced form Rb^+^TCNQ^•−^ (ρ = −1).^41^ Figure 2b plots the estimated ρ values at respective temperatures for **1‐DCE**, indicating that the TCNQ(EtO)_2_ moiety exhibits a quinonoid structure (N‐form) at room temperature, whereas it changes into a quasibenzenoid form (I‐form) on cooling at 230 K. Thus, the variation in the charge of TCNQ(EtO)_2_ moiety is completely consistent with that observed for the [Ru_2_] unit, confirming the occurrence of the one‐step N–I phase transition at 230 K.

### 2.3 Temperature‐Dependent Infrared Spectra of **1‐DCE**

**Figure 3:**
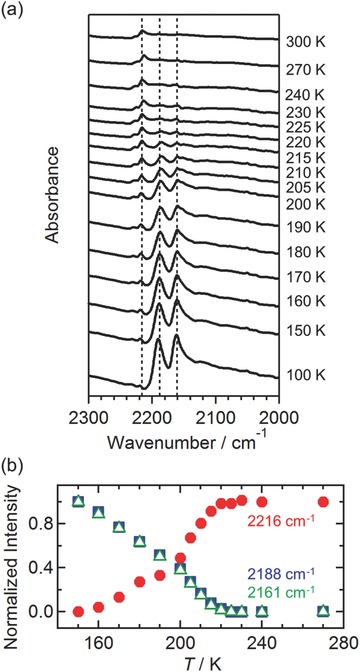
a) Infrared absorption spectra of **1‐DCE** at wavenumbers ranging from 2000 to 2300 cm^−1^ measured using a Nujol mull, wherein the band at 2216 cm^−1^ corresponds to the ν(C≡N) of neutral TCNQ(EtO)_2_ moieties and the bands at 2161 and 2188 cm^−1^ correspond to the ν(C≡N) of ionic TCNQ(EtO)_2_ moieties. b) Temperature dependence of peak intensities.

The N–I phase transition in **1‐DCE** was additionally confirmed by infrared absorption spectroscopy. Characteristic C≡N stretching modes (ν_C≡N_) of TCNQ(EtO)_2_ were observed at frequencies ranging from 2100 to 2250 cm^−1^ (**Figure** **3**a; heating process). At temperatures 300–230 K, ν_C≡N_ was observed as a single band at 2216 cm^−1^ corresponding to the N‐state of TCNQ(EtO)_2_ (e.g., 2221 cm^−1^ for free TCNQ(EtO)_2_). However, at temperatures less than 225 K, two new bands were observed at 2188 and 2161 cm^−1^, corresponding to the characteristic ν_C≡N_ for the I‐state of TCNQ(EtO)_2_^•−^ (e.g., 2199 and 2172 cm^−1^ for the anion radical salt Li^+^TCNQ(EtO)_2_^•−^). The variation in the temperature of the normalized peak intensities for the respective peaks exhibited an inflection point at 220–225 K (Figure 3b). This temperature variation is in good agreement with that observed for structures. A similar trend was observed for the C=C stretching mode (ν_C=C_) (Figure S2, Supporting Information).

### 2.4 Effect of the N–I Phase Transition on the Magnetic Properties of **1‐DCE**

**Figure 4:**
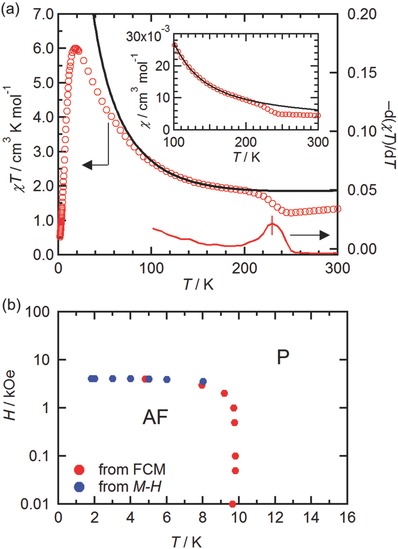
a) Temperature dependence of *χT* and −d*χT*/d*T* for **1‐DCE** measured under a DC field of 1 kOe. Inset shows the temperature dependence of χ for temperatures ranging from 100 to 300 K. The solid line represents the best‐fit line with an alternating chain model of *S_i_* = 3/2 and *S_i_*_+1_ = 1/2 for the data at temperatures ranging from 110 to 190 K. b) *H*–*T* phase diagrams of **1‐DCE** in the low‐temperature region.

The most notable feature of **1‐DCE** is that a ferrimagnetic 1D correlation can be observed between the I‐phase of **1‐DCE** with *S* = 3/2 for [Ru_2_^II,III^]^+^ and *S* = 1/2 for TCNQ(EtO)_2_^•−^, whereas the N‐phase is paramagnetic with *S* = 1 for [Ru_2_^II,II^]. The *χT* product of **1‐DCE** at 300 K was 1.34 cm^3^ K mol^−1^. This value is in good agreement with the typical value observed for the isolated [Ru_2_^II,II^] species with *S* = 1.^16^, ^17^, ^42^, ^43^, ^44^, ^45^ With decreasing temperature, the *χT* product monotonically decreased because of the effect of the anisotropic nature of [Ru_2_^II,II^];^16^, ^17^, ^42^, ^43^, ^44^, ^45^ however, it suddenly increased from 1.22 cm^3^ K mol^−1^ at 250 K to 1.87 cm^3^ K mol^−1^ at 200 K, and gradually to 6.01 cm^3^ K mol^−1^ at 18 K, followed by an abrupt decrease to 0.52 cm^3^ K mol^−1^ at 1.8 K (**Figure** **4**a). In addition, this sudden shift of *χT* can be observed in the χ–*T* plot (see inset of Figure 4a), indicating the occurrence of the N–I phase transition with a magnetic change from isolated paramagnetic spins to ferrimagnetically correlated spins. By taking d(*χT*)/d*T* (peak top), *T*_c_ was determined to be 230 K (Figure 4a). The χ and *χT* data were simulated at temperatures ranging from 110 to 190 K using an alternating chain model with *S_i_* = 3/2 and *S_i_*_+1_ = 1/2 in the Hamiltonian *H* = −2*J*Σ*^N^_i_*_=1_ *S_i_·S_i_*_+1_,^46^ and an adequate parameter set was obtained as *g*_[Ru2]_ = 2.181(2); *g*_Rad_ = 2.0 (fix); and *J*/*k*_B_ = −105.2(6) K (Figure 4a). The spins of [Ru_2_^II,III^]^+^ and TCNQ(EtO)_2_^•−^ were strongly antiferromagnetically coupled, and the magnitude of *J* was in good agreement with that reported previously for chain compounds.^11^, ^47^ The strong antiferromagnetic coupling can be caused by a considerable overlap between the frontier π* singly occupied molecular orbital (SOMO) of [Ru_2_^II,III^]^+^ and the π* SOMO of TCNQ(EtO)_2_^•−^. The 1D correlated spins eventually led to antiferromagnetic long‐range ordering at *T_N_* = 9.6 K related to the presence of the interchain antiferromagnetic interactions (vide infra), similar to the ionic D^+^A^−^ chain, [Ru_2_(4‐Cl‐2‐MeOPhCO_2_)_4_(BTDA‐TCNQ)]·*n*(solv).^47^ This result was confirmed by the AC susceptibility data (Figure S3a, Supporting Information); an *H*–*T* phase diagram (Figure 4b) was constructed using the data obtained from the field‐cooled magnetization (FCM) measured at several DC fields (Figure S3b, Supporting Information) and the field dependence of the magnetization measured at several temperatures (Figure S3c, Supporting Information). Using the mean‐field approximation with an effective spin *S*_eff_ = *S*_[Ru2]_ −*S*_Rad_ = 1, the interchain interaction was found to be *zJ′*/*k*_B_ = −0.27 K based on the equation 2|*zJ′*|*S*_eff_^2^ ≈ *gS*_eff_μ_B_ *H*_ex_, with a spin‐flipping field *H*_ex_ = 0.40 T at 1.8 K and *g* = 2 (Figure S3c, Supporting Information)^48^ although *zJ′* was only useful for comparison.

### 2.5 Characterization of Desolvated Compound **1**

**Figure 5:**
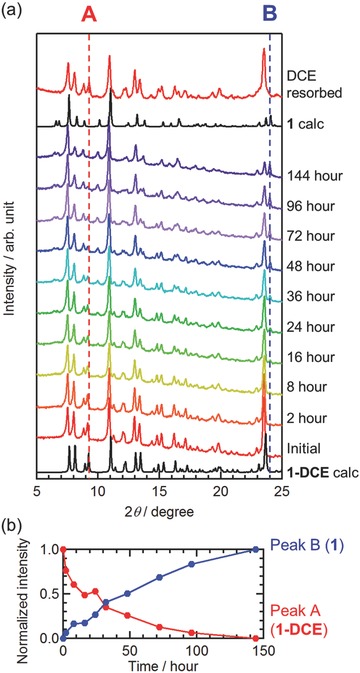
Time‐course variations in the powder X‐ray diffraction (PXRD) pattern of **1‐DCE** with evacuation at 298 K. a) PXRD patterns and b) time dependence of the peak A for **1‐DCE** (in red) and peak B for **1** (in blue).

Compound **1‐DCE** was relatively stable even at room temperature compared with the first N–I transition compound;^11^ however, it gradually released the interstitial DCE molecules with increasing temperature under N_2_, thereby affording DCE‐free compound (**1**). Compound **1** was considerably stable at high temperatures up to 450 K and simultaneously maintained its crystallinity as evidenced by thermogravimetric analysis (TGA) (Figure S4, Supporting Information). In addition, **1** could be prepared by evacuation at room temperature. Time‐course measurements for room temperature powder X‐ray diffraction patterns (PXRDs) under vacuum demonstrated that the structure undergoes a gradual, continuous modification over several days (**Figure** **5**). Most of the diffraction peaks for **1** were almost identical with those in **1‐DCE**, whereas some new peaks were observed for **1**, leading to twice the cell volume (see below). The structure of **1** was considered to be a superstructure of **1‐DCE**.

**Figure 6:**
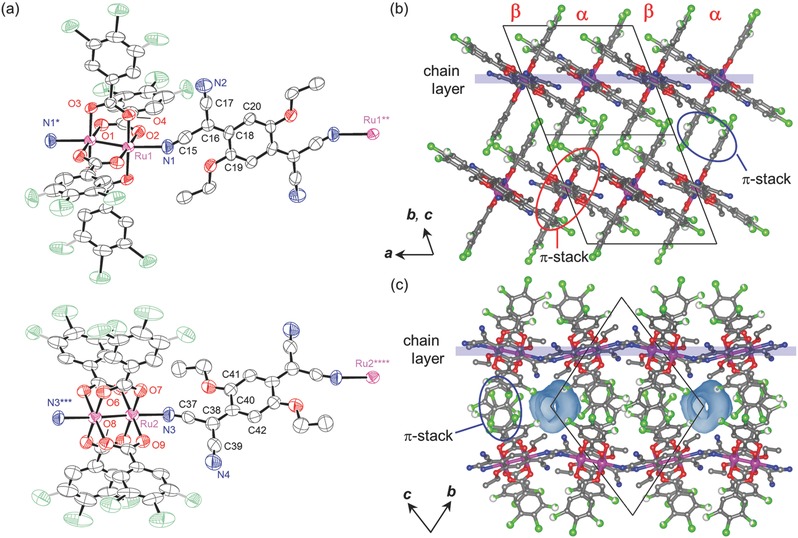
Crystal structure of **1** at 103 K. a) Thermal ellipsoid plot showing the formula unit and atom numbering scheme for α‐ (top) and β‐units (bottom). Displacement ellipsoids are drawn at a 50% probability level. Gray bond indicates the minor component of the positional disorder: Packing diagrams projected along the b) <01−1> direction (chain‐running direction) and c) *a* axis (side view of the chain layer). Blue, red, gray, green, and purple represent N, O, C, F, and Ru, respectively. Hydrogen atoms are omitted for clarity. Connolly surfaces are also depicted in (c).

Although the unit cell contained two crystallographically unique DA pairs (distinguished hereafter as α‐ and β‐pairs), **1** also crystallized in the triclinic *P*‐1 space group (Tables S1–S5, Supporting Information), wherein the inversion centers were located at the midpoint of the Ru—Ru bond and center of the TCNQ(EtO)_2_ moiety in the respective DA pairs (*Z* = 2, **Figure** **6**a).^49^ Hence, two crystallographically unique chains, α‐ and β‐chains, exist in a crystal. Both the chains ran along the <01−1> direction to form a chain layer on the (011) plane (Figure 6b,c). Within the chain layer, the α‐ and β‐chains were alternately arranged in an antiphase manner (i.e., ⋅⋅⋅D^α^⋅⋅⋅A^β^⋅⋅⋅D^α^⋅⋅⋅A^β^⋅⋅⋅ or ⋅⋅⋅A^α^⋅⋅⋅D^β^⋅⋅⋅A^α^⋅⋅⋅D^β^⋅⋅⋅) along the <100> direction with an interchain π‐stack as observed for **1‐DCE** (red circles in Figure 6b), leading to a shorter [Ru_2_]⋅⋅⋅TCNQ distance of 6.82 Å at 103 K (centroid to centroid) compared with that in **1‐DCE**. 1D void space channels with a solvent‐accessible volume of 159 Å^3^ (6.4%) at 103 K were retained between the chain layers after the release of the DCE molecules from **1‐DCE**, which ran along the <100> direction, i.e., perpendicular to the chain‐running direction (Figure 6c shows the Connolly surfaces). Thus, the interchain layers aligned in an in‐phase manner along the <011> direction (i.e., ⋅⋅⋅D^α^⋅⋅⋅D^β^⋅⋅⋅D^α^⋅⋅⋅D^β^⋅⋅⋅ or ⋅⋅⋅A^α^⋅⋅⋅A^β^⋅⋅⋅A^α^⋅⋅⋅A^β^⋅⋅⋅) are significantly separated by 11.71 Å at 103 K (Figure 6c) although the π‐stacks are still present between the benzoate groups (blue circles in Figure 6b). Thus, the orientation of DA unit is slightly modified by the desolvation for canceling the vacancy originally made by DCE in **1‐DCE**. Since the chain layer comprised compact chains, only half of the void space canceled without any significant change in the interchain relationship.

Temperature variation was the most important aspect in the structure of **1**. The average Ru—O_eq_ bond lengths for the α‐ and β‐chains in **1** were in the range of [Ru_2_^II,II^] in the entire temperature range investigated (103–270 K) (Table S3, Supporting Information). Corresponding to the oxidation state of [Ru_2_^II,II^], the charge of the TCNQ(EtO)_2_ moieties corresponded to the neutral form at all the investigated temperatures (Table S4, Supporting Information). Hence, the α‐ and β‐chains in **1** are in the N‐state at *T* ≥ 103 K, which is completely different from the observation of the N–I phase transition in **1‐DCE**. This result was confirmed by the temperature variation of infrared (IR) spectra of **1** (Figure S5, Supporting Information).

### 2.6 In Situ Magnetic Measurements for the Variation from **1‐DCE** to **1**

**Figure 7:**
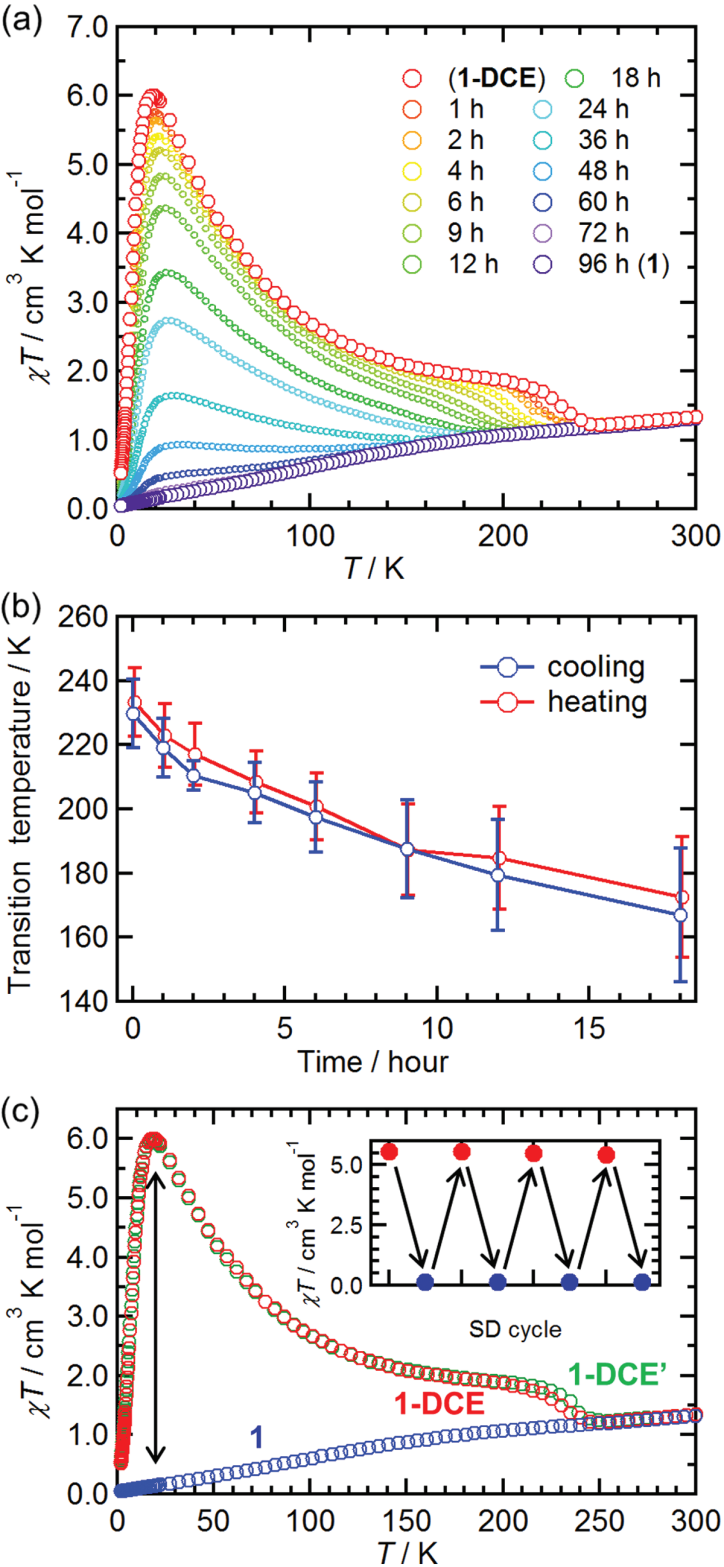
Time‐course magnetic variation of **1‐DCE**. a) *χT*–*T* plots measured at 1 kOe. b) Time dependence of the N–I transition temperature defined by the temperature at which a peak appears in the −d*χT*/d*T* versus *T* plots. Error bar indicates the temperature width for the N–I phase transition defined by the half‐width of the peak in the −d*χT*/d*T* versus *T* plots. c) Variation in the *χT*–*T* plots measured at 1 kOe by the transformation between **1‐DCE** and **1** by desorption/sorption (SD) cycles. Inset shows the *χT* value at 18 K for each step.

A microcrystal sample of **1‐DCE** in a gelatin capsule was inserted into an MPMS‐7X system (Quantum Design Ltd), and in situ magnetic measurements with stepwise desolvation treatment were conducted. After the measurement of the fresh **1‐DCE** sample (Figure 4), it was desolvated in situ by evacuation for a designated time at 310 K. After evacuation, the cell was filled with He gas to enhance the thermal conductivity, and the FCM under a field of 1 kOe was recorded from 300 K (**Figure** **7**a). With desolvation, the *T*_c_ continuously shifted to lower temperatures than the original *T*_c_ = 230 K (Figure 7b) although the temperature width for the N–I transition slightly increased (obtained from the d*χT*/d*T*–*T* plot; Figure S6, Supporting Information),^50^ and the peaks in the *χT*–*T* plots certainly decreased. After evacuating for 18 h, the *T*_c_ was no longer detected from the d*χT*/d*T*–*T* plot because of the absence of peaks (Figure S6, Supporting Information). The peak in the χ–*T* plots (Figure S7a, Supporting Information) depicted that the desolvation gradually progressed after the 18 h evacuation. However, it was almost completed after the evacuation for 96 h (Figure S7b, Supporting Information); the final *χT*–*T* feature was observed for **1** with the isolated [Ru_2_^II,II^] species. The magnetic behavior of **1** was interpreted as a typical case for the [Ru_2_^II,II^] species with *S* = 1 considering the zero‐field splitting (*D*), the temperature‐independent paramagnetism (χ_TIP_), and an impurity with *S* = 3/2(ρ_imp_) (Figure S8, Supporting Information).

### 2.7 Resolvation for **1**

Desolvated compound **1** completely reverted to **1‐DCE** by the resolvation of DCE. With the exposure of **1** to a DCE vapor for 72 h at 298 K in a sealed MPMS cell, the magnetic behavior of the compound (denoted as **1‐DCE′**) was essentially the same as that of the original **1‐DCE** (Figure 7c and Figure S9 (Supporting Information)). These results indicated that both **1‐DCE** and **1** undergo excellent reversibility between a spin‐correlated system with the N–I phase transition (**1‐DCE**) and a paramagnetic system (**1**) by the application of SD treatments; a magnetic switch via SD cycles was demonstrated (inset of Figure 7c).

A DCE vapor adsorption isotherm at 298 K revealed that **1** adsorbs ≈1 mole of DCE (Figure S10, Supporting Information), which is consistent with the formula of **1‐DCE**. This reversibility can be confirmed by the structural change; the PXRD pattern of **1** changed to its original pattern for **1‐DCE** by exposing **1** to DCE vapor for 24 h (Figure 5a).

### 2.8 Tuning the N–I Phase Transition by Applying Pressure

The pressure variation of the N–I phase transitions of **1‐DCE** was investigated by applying hydrostatic pressure using a piston‐cylinder‐type cell made of CuBe alloy in a SQUID magnetometer. Figure S11a–c (Supporting Information) shows the temperature dependence of the magnetization under various applied pressures, and Figure S11d (Supporting Information) shows the diagram of pressure versus *T*. The *T*_c_ was dependent on the hydrostatic pressure. Even at low pressures of less than 1 kbar, *T*_c_ sensitively shifted to high temperature and exceeded the upper limit of the measurement temperature range. We also applied pressures up to 10.5 kbar to **1**, though, magnetic properties remained unchanged.

### 2.9 Solvation‐Modified N–I Transition

The electronic state of simple charge‐transfer complexes is approximately represented as follows^51^

(1) $$E=\left(I_{D}-E_{A}\right)-\alpha V$$

where *V* is the Coulombic attractive force (>0) between the nearest DA pairs, and α is the Madelung constant depending on the crystal structure. The N‐ and I‐ phases were expected in the regimes (*I*_D_ − *E*_A_) > *αV* and (*I*_D_ − *E*_A_) < *αV*, respectively, and the N–I transformation was expected to occur at (*I*_D_ − *E*_A_) = *αV*. As *I*_D_ and *E*_A_ are the basic intrinsic parameters for D and A, respectively, the (*I*_D_ − *E*_A_) value could be the same between **1‐DCE** and **1**. In contrast, the Madelung energy *αV* was extremely sensitive to structural changes, i.e., the orbital overlap between the D and A units; with the increasing overlap, the *αV* value tended to increase and vice versa.^1^ The application of hydrostatic pressures to **1‐DCE**, which possibly increased the orbital overlap between the D and A units, and the distance between D and A, promoted the shift of the N–I transition boundary to high temperatures (Figure S11d, Supporting Information). In particular, the orbital overlap in **1** has been proposed to decrease from that in **1‐DCE** via desolvation. A simple structural comparison between **1‐DCE** and **1** revealed that the chain form of **1‐DCE** is considerably more linear than that of **1**, indicative of a larger overlap between the π* orbitals of TCNQ(EtO)_2_ and the [Ru_2_] core for **1‐DCE**. To evaluate this hypothesis, an angular overlap model was considered (Table S3, Supporting Information). The angular part of π components (*A*_π_) that can overlap between [Ru_2_^II,II^] π* and TCNQ(EtO)_2_π* was expressed as *A*_π_ = {1 − (sinδ·sinτ)^2^}^0.5^, where δ represents the Ru—N—C angle and τ represents the dihedral angle between the least‐square planes composed of C—C—(C≡N)_2_ and Ru—Ru—N≡C—C (Table S3, Supporting Information).^52^ The crystal structure for **1‐DCE** at 270 K (N‐phase) exhibited *A*_π_ = 1.00, whereas the structure for **1** at 270 K exhibited *A*_π_ = 0.95 and 0.97 for α‐ and β‐chain, respectively (Table S3, Supporting Information). Compound **1** exhibited a less‐overlapped form. The structural deformation induced by the desolvation slightly weakened the CT interactions in **1**, leading to the suppressed N–I phase transition. As described above, even a subtle structural difference is crucial for the observation of the N–I phase transition in covalently bonded DA chain systems, possibly explaining the inability of applied pressure to induce the N–I phase transition in **1** although **1‐DCE** is extremely sensitive to pressure (Figure S11d, Supporting Information).

## 3 Conclusion

In this study, **1‐DCE** is the first example of a one‐step N–I phase transition among the two examples of this type of DA chain complexes that exhibit the N–I phase transition. Furthermore, the present case is a new type of N–I phase transition system in which the *T*_c_ is continuously varying depending on the accommodation degree of crystallization solvents; completely desolvated compound **1** is eventually a simple N‐state system. These results suggest that the electronic state of partially desolvated compounds is not simply represented by the summation of the abundance ratios of **1‐DCE** and **1**, although the N‐ (DA) and I‐domains (D^+^A^−^) coexist in the partially desolvated compounds as derived from their PXRD patterns, wherein the intensity of the diffraction peaks for **1** compensates for that for **1‐DCE** (Figure 5b). As mentioned above, the N–I phase transition is sensitively affected by the environment in which the D and A units are located because of the Coulomb interactions and structural modifications. Hence, a partial desolvation could induce a DA condition variable at each step of the desolvation process. A key for such continuous regulation is the structural similarity between **1‐DCE** and **1**, although even the marginal structural deformation significantly affects the N–I phase transition behavior. However, the control of the N–I phase transition via an external chemical stimulus first demonstrated in the present system will offer a new methodology for the design of switchable materials.

## 4 Experimental Section

*General Procedures and Materials*: All synthetic procedures were performed under inert atmosphere using standard Schlenk‐line techniques and a commercial glove box. All chemicals purchased from commercial sources were of reagent‐grade quality. Solvents were distilled under N_2_ using common drying agents. [Ru_2_(3,4‐Cl_2_PhCO_2_)_4_(THF)_2_] as precursors for **1‐DCE** were synthesized according to previously reported methods.^16^

*Preparation of* ***1‐DCE***: First, a solution of TCNQ(EtO)_2_ (23.4 mg, 0.08 mmol) in CH_2_Cl_2_ (40 mL) was separated into 20 portions and added into glass tubes with a narrow diameter of φ8 mm (bottom layer). Second, a mixed solvent of CH_2_Cl_2_/C_2_H_4_Cl_2_ in a volume ratio of 1:1 (1 mL) was added on the bottom layer for decreasing the diffusion rate (middle layer). Finally, [Ru_2_(3,4‐Cl_2_PhCO_2_)_4_(THF)_2_] (88.5 mg, 0.08 mmol) in C_2_H_4_Cl_2_ (40 mL) was carefully added on the middle layer of each tube (top layer). The glass tubes were left undisturbed for one month to obtain block‐type brown crystals of **1‐DCE** (24% yield). IR (Nujol): ν = 2216 (s; ν(C≡N)), 2188 (s; ν(C≡N)), 2161 cm^−1^ (s; ν(C≡N)); Anal. calcd. for C_46_H_28_Cl_10_N_4_O_10_Ru_2_: C 40.82, H 2.08, N 4.14; found: C 40.47, H 1.98, N 4.28.

*Preparation of* ***1***: **1‐DCE** was heated to 353 K under vacuum for 24 h. IR (Nujol): ν = 2214 (s; ν(C≡N)), 2189 (s; ν(C≡N)), 2150 cm^−1^ (s; ν(C≡N)); Anal. calcd. for C_44_H_24_Cl_8_N_4_O_10_Ru_2_: C 42.12, H 1.93, N 4.47; found: C 41.89, H 2.10, N 4.49.

*Physical Measurements*: IR spectra were obtained on a Jasco FT‐IR 620 spectrometer using Nujol mulls sandwiched between CaF_2_ plates. TGA was performed on a Shimadzu DTG‐60H apparatus under N_2_ at temperatures ranging from 298 to 673 K at a heating rate of 5 K min^−1^. PXRD patterns were obtained using an Ultima IV diffractometer with Cu *Kα* radiation (λ = 1.5418 Å) at room temperature.

*X‐Ray Crystallographic Analyses of* ***1‐DCE*** *and* ***1***: Crystal data for **1‐DCE** and **1** were recorded at 103–270 K (heating process) on a charge‐coupled device (CCD) diffractometer (Rigaku Saturn 724) with multilayer mirror monochromated Mo Kα radiation (λ = 0.71075 Å). A single crystal was mounted on a thin Kapton film using Nujol and cooled under N_2_. The structures were solved using direct methods (SHELXT Version 2014/5),^53^ which were expanded using Fourier techniques. All calculations were performed using the Crystal Structure crystallographic software package,^54^ except for refinement, which was performed using SHELXL Version 2014/7.^55^ CCDC 1565753 and 1565754 contain the supplementary crystallographic data for **1‐DCE** at 103 K and for **1** at 103 K, respectively. These data can be obtained free of charge from The Cambridge Crystallographic Data Centre (CCDC) via http://www.ccdc.cam.ac.uk/data_request/cif. Please contact the corresponding author if the structural information under the temperatures other than 103 K as crystallographic information file (CIF) format is required. Structural diagrams were prepared using the VESTA software.^56^ The void volumes of crystal structures were estimated by PLATON.^57^

*Vapor Sorption Measurements*: Sorption isotherm measurements for DCE were performed using an automatic volumetric adsorption apparatus (BELSORP‐max; BEL Inc.) at 298 K. A known weight (≈50 mg) of the dried sample was placed into the sample cell, and prior to measurements, the sample cell was evacuated again using the degas function of the analyzer for 12 h at 353 K. Finally, the change in the pressure was monitored, and the degree of adsorption was determined by the decrease in the pressure at the equilibrium state.

*In Situ Vapor Desorption–Magnetic Measurements*: Magnetic susceptibility measurements were conducted on a Quantum Design (SQUID) magnetometer MPMS‐XL using a polycrystalline sample at the temperatures ranging from 1.8 to 300 K at 1000 Oe. The data were corrected for the diamagnetic contribution of the sample using Pascal's constants.^58^ A known weight (≈25 mg) of the dried sample was placed in a gelatin capsule, and the capsule was held at the center of a plastic straw. The straw was then attached on the edge of a homemade sample rod made of a SUS tube and a brass male thread with a fluorocarbon tape. The sample was isolated from the surrounding atmosphere by overlaying a close‐ended brass tube, which can be attached to the thread on the open end of the sample rod using screws. An airtight seal between the thread and brass tube was achieved using a silicon sealant (CAF4, Bluestar Silicones). The SUS tube was connected to a gas handling system with a turbomolecular pump and manometer. The background signals need not be subtracted from the brass tube. The sample was evacuated at 310 K for a designated time (Figure 7a). He was introduced at a pressure of 100 kPa before starting the measurement.

*Magnetic Measurements under Applied Pressure*: Pressure was applied to polycrystalline samples of **1‐DCE** in Apiezon‐J oil (as the pressure transmitting medium) using a piston‐cylinder‐type cell made of a Cu–Be alloy. The cell was placed in a SQUID magnetometer, and the N–I phase transitions were examined by monitoring magnetic changes; the actual pressure was estimated from the superconducting transition temperature of Pb.

## Conflict of Interest

The authors declare no conflict of interest.

## Supporting information

Supplementary

## References

[1] G. Saito , Y. Yoshida , Bull. Chem. Soc. Jpn. 2007, 80, 1.

[2] N. Toyota , M. Lang , J. Müller , Low‐Dimensional Molecular Metals, Springer, Heidelberg, Germany 2007.

[3] A. Graja , Low‐Dimensional Organic Conductors, World Scientific, Singapore 1992.

[4] T. Enoki , T. Miyazaki , Chem. Rev. 2004, 104, 5449. 15535656 10.1021/cr0306438

[5] E. Coronado , P. Day , Chem. Rev. 2004, 104, 5419. 15535655 10.1021/cr030641n

[6] J. Hubbard , J. B. Torrance , Phys. Rev. Lett. 1981, 47, 1750.

[7] P. Bak , Rep. Prog. Phys. 1982, 45, 587.

[8] R. Bruinsma , P. Bak , J. B. Torrance , Phys. Rev. B 1983, 27, 456.

[9] J. B. Torrance , J. E. Vazquez , J. J. Mayerle , V. Y. Lee , Phys. Rev. Lett. 1981, 46, 253.

[10] J. B. Torrance , A. Girlando , J. J. Mayerle , J. I. Crowley , V. Y. Lee , P. Batail , S. LaPlaca , Phys. Rev. Lett. 1981, 47, 1747.

[11] H. Miyasaka , N. Motokawa , T. Chiyo , M. Takemura , M. Yamashita , H. Sagayama , T. Arima , J. Am. Chem. Soc. 2011, 133, 5338. 21410157 10.1021/ja110007u

[12] K. Nakabayashi , H. Miyasaka , Chem. ‐ Eur. J. 2014, 20, 5121. 24623620 10.1002/chem.201304420

[13] K. Nakabayashi , M. Nishio , H. Miyasaka , Inorg. Chem. 2016, 55, 2473. 26878151 10.1021/acs.inorgchem.5b02858

[14] H. Miyasaka , Acc. Chem. Res. 2013, 46, 248. 23128042 10.1021/ar300102t

[15] W. Kosaka , T. Morita , T. Yokoyama , J. Zhang , H. Miyasaka , Inorg. Chem. 2015, 54, 1518. 25629292 10.1021/ic502513p

[16] W. Kosaka , M. Itoh , H. Miyasaka , Dalton Trans. 2015, 44, 8156. 25847192 10.1039/c5dt00505a

[17] H. Miyasaka , N. Motokawa , R. Atsuumi , H. Kamo , Y. Asai , M. Yamashita , Dalton Trans. 2011, 40, 673. 21135952 10.1039/c0dt00956c

[18] W. Kosaka , K. Yamagishi , H. Yoshida , R. Matsuda , S. Kitagawa , M. Takata , H. Miyasaka , Chem. Commun. 2013, 49, 1594. 10.1039/c2cc36153a 23174816

[19] W. Kosaka , K. Yamagishi , A. Hori , H. Sato , R. Matsuda , S. Kitagawa , M. Takata , H. Miyasaka , J. Am. Chem. Soc. 2013, 135, 18469. 24151906 10.1021/ja4076056

[20] W. Kosaka , K. Yamagishi , R. Matsuda , S. Kitagawa , M. Takata , H. Miyasaka , Chem. Lett. 2014, 43, 890.

[21] J. Zhang , W. Kosaka , H. Fukunaga , S. Kitagawa , M. Takata , H. Miyasaka , Inorg. Chem. 2016, 55, 12085. 27934304 10.1021/acs.inorgchem.6b02349

[22] C. Dou , W. Kosaka , H. Miyasaka , Chem. Lett. 2017, 46, 1288.

[23] V. Niel , A. L. Thompson , M. C. Muñoz , A. Galet , A. E. Goeta , J. A. Real , Angew. Chem. Int. Ed. 2003, 42, 3760. 10.1002/anie.200351853 12923837

[24] G. J. Halder , K. W. Chapman , S. M. Neville , B. Moubaraki , K. S. Murray , J. F. Létard , C. J. Kepert , J. Am. Chem. Soc. 2008, 130, 17552. 19053411 10.1021/ja8068038

[25] M. Ohba , K. Yoneda , G. Agusti , M. C. Muñoz , A. B. Gaspar , J. A. Real , M. Yamasaki , H. Ando , Y. Nakao , S. Sakaki , S. Kitagawa , Angew. Chem. Int. Ed. 2009, 48, 4767. 10.1002/anie.200806039 19294711

[26] W. Kosaka , K. Yamagishi , J. Zhang , H. Miyasaka , J. Am. Chem. Soc. 2014, 136, 12304. 25120189 10.1021/ja504992g

[27] A. A. Talin , A. Centrone , A. C. Ford , M. E. Foster , V. Stavila , P. Haney , R. A. Kinney , V. Szalai , F. E. Gabaly , H. P. Yoon , F. Léonard , M. D. Allendorf , Science 2013, 1246738. 10.1126/science.1246738 24310609

[28] D. Maspoch , D. Ruiz‐Molina , J. Veciana , Chem. Soc. Rev. 2007, 36, 770. 17471401 10.1039/b501600m

[29] P. Dechambenoit , J. R. Long , Chem. Soc. Rev. 2011, 40, 3249. 21298169 10.1039/c0cs00167h

[30] E. Coronado , G. M. Espallargas , Chem. Soc. Rev. 2013, 42, 1525. 23146915 10.1039/c2cs35278h

[31] D. Maspoch , D. Ruiz‐Molina , K. Wurst , N. Domingo , M. Cavallini , F. Biscarini , J. Tejada , C. Rovira , J. Veciana , Nat. Mater. 2003, 2, 190. 12612678 10.1038/nmat834

[32] S. Ohkoshi , K. Arai , Y. Sato , K. Hashimoto , Nat. Mater. 2004, 3, 857. 15558035 10.1038/nmat1260

[33] N. Motokawa , S. Matsunaga , S. Takaishi , H. Miyasaka , M. Yamashita , K. R. Dunbar , J. Am. Chem. Soc. 2010, 132, 11943. 20701282 10.1021/ja102412g

[34] J. Larionova , S. A. Chavan , J. V. Yakhmi , A. G. Frøystein , J. Sletten , C. Sourisseau , O. Kahn , Inorg. Chem. 1997, 36, 6374.

[35] G. J. Halder , C. J. Kepert , B. Moubaraki , K. S. Murray , J. D. Cashion , Science 2002, 298, 1762. 12459583 10.1126/science.1075948

[36] According to IUCr rule, a set of lattice constants where all angles are acute or obtuse angles should be employed in the triclinic cell among all the possible set of lattice constants. In **1‐DCE**, angle β is a bit smaller than 90° at 103 K, which exceeds 90° with the increase of temperature.

[37] M. Nishio , N. Hoshino , W. Kosaka , T. Akutagawa , H. Miyasaka , J. Am. Chem. Soc. 2013, 135, 17715. 24164661 10.1021/ja409785a

[38] F. A. Cotton , C. A. Murillo , R. A. Walton , Multiple Bonds between Metal Atoms, 3rd ed., Springer, New York 2005.

[39] T. J. Kistenmacher , T. J. Emge , A. N. Bloch , D. O. Cowan , Acta Crystallogr., Sect. B: Struct. Sci., Cryst. Eng. Mater. 1982, 38, 1193.

[40] R. E. Long , R. A. Sparks , K. N. Trueblood , Acta Crystallogr. 1965, 18, 932.

[41] C. J. Fritsche Jr. , P. Arthur Jr. , Acta Crystallogr. 1966, 21, 139.

[42] P. Maldivi , A.‐M. Giroud‐Godquin , J.‐C. Marchon , D. Guillon , A. Skoulios , Chem. Phys. Lett. 1989, 157, 552.

[43] F. A. Cotton , V. M. Miskowski , B. Zhong , J. Am. Chem. Soc. 1989, 111, 6177.

[44] L. Bonnet , F. D. Cukiernik , P. Maldivi , A.‐M. Giroud‐Godquin , J.‐C. Marchon , Chem. Mater. 1994, 6, 31.

[45] E. V. Dikarev , A. S. Filatov , R. Clérac , M. A. Petrukhina , Inorg. Chem. 2006, 45, 744. 16411710 10.1021/ic051537m

[46] M. Drillon , E. Coronado , D. Beltran , R. Georges , Chem. Phys. 1983, 79, 449.

[47] M. Nishio , H. Miyasaka , Inorg. Chem. 2014, 53, 4716. 24750071 10.1021/ic500413j

[48] R. L. Carlin , Magnetochemistry, Springer‐Verlag, Berlin, Germany 1986.

[49] The cell system for **1‐DCE** at 103 K can be transformed into the corresponding one for **1** by the following relationships; ***a′*** = −***a*** + ***b***, ***b′*** = ***b*** − ***c***, and ***c′*** = −***a*** − ***c***, with *V′* = 2*V* where ***a***, ***b***, and ***c*** are the original axis vectors in **1‐DCE** at 103 K, and ***a′***, ***b′***, and ***c′*** are the transformed ones. *V* and *V′* indicate the cell volume under the original cell system and the transformed one, respectively.

[50] The temperature width for the N–I transition was defined by the half width of the peak in the −d*χT*/d*T*–*T* plots for the determination of *T* _c_.

[51] H. M. McConnell , B. M. Hoffman , R. M. Metzger , Proc. Natl. Acad. Sci. USA 1965, 53, 46. 16578588 10.1073/pnas.53.1.46 PMC219431

[52] Y. Sayama , M. Honda , M. Mikuriya , I. Hiromitsu , K. Kasuga , Bull. Chem. Soc. Jpn. 2003, 76, 769.

[53] G. M. Sheldrick , Acta Crystallogr. 2014, A70, C1437.

[54] CrystalStructure 4.2.1: Crystal Structure Analysis Package, Rigaku Corp, Tokyo, Japan **2000–2016**.

[55] G. M. Sheldrick , Acta Crystallogr. 2008, A64, 112.

[56] K. Momma , F. Izumi , J. Appl. Crystallogr. 2008, 41, 653.

[57] A. L. Spek , Acta Crystallogr., Sect. A: Found. Adv. 1990, 46, 194.

[58] E. A. Boudreaux , L. N. Mulay , Theory and Applications of Molecular Paramagnetism, John Wiley & Sons, New York 1976.

