# Supplementary material for: Magnetic Sponge with Neutral–Ionic Phase Transitions
Source: Adv Sci (Weinh). 2017 Dec 4;5(2):1700526. doi: 10.1002/advs.201700526 (PMC5827013; doi:10.1002/advs.201700526)
Supplement: Supplementary file 1 — Supplementary [file ADVS-5-1700526-s001.pdf]

Copyright WILEY-VCH Verlag GmbH & Co. KGaA, 69469 Weinheim, Germany, 2017.

ADVANCED SCIENCE Open Access

Supporting Information

for *Adv. Sci.,* DOI: 10.1002/advs.201700526

Magnetic Sponge with Neutral–Ionic Phase Transitions

*Wataru Kosaka, Yusuke Takahashi, Masaki Nishio, Keisuke Narushima, Hiroki Fukunaga, and Hitoshi Miyasaka**

Copyright WILEY-VCH Verlag GmbH & Co. KGaA, 69469 Weinheim, Germany, 2016.

Supporting Information

Magnetic Sponge with Neutral–Ionic Phase Transitions

*Wataru Kosaka, Yusuke Takahashi, Masaki Nishio, Keisuke Narushima, Hiroki Fukunaga, and Hitoshi Miyasaka**

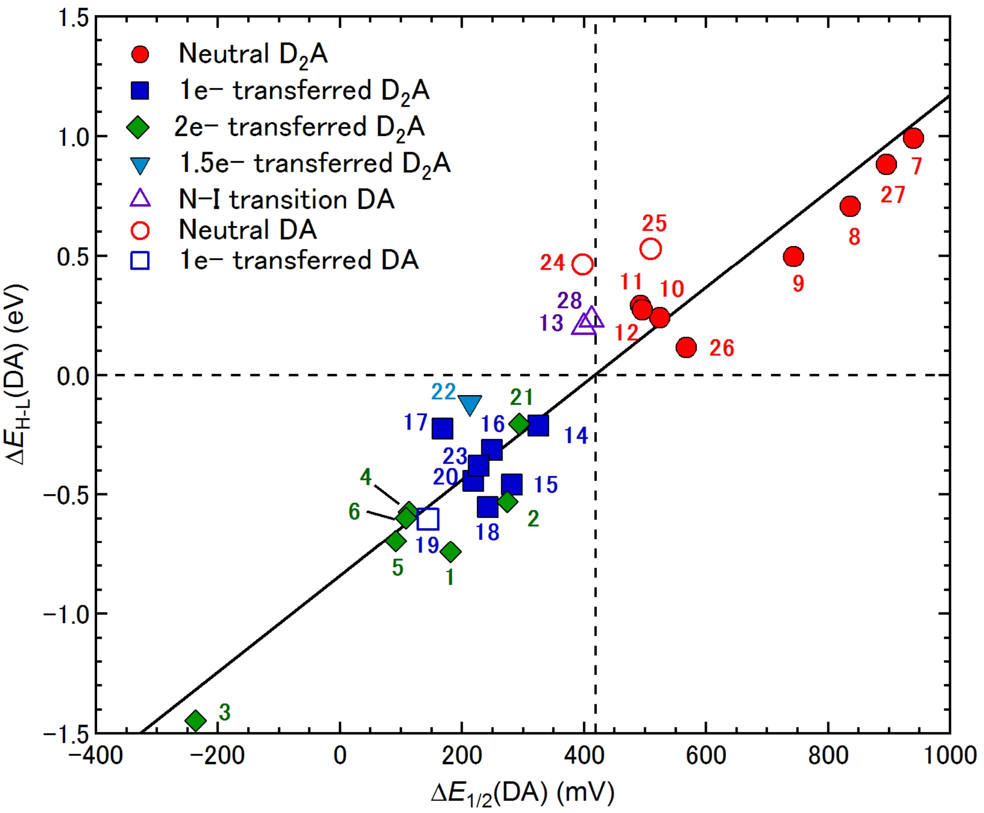

Figure S1. Plot of $\Delta E_{H\text{-}L}$(DA) vs $\Delta E_{1/2}$(DA) for D_2A and DA materials of $[Ru_2^{II,II}]$/TCNQR$_x$, DCNQIR$_x$ sets reported until now, where red circle, blue square, green diamond and cyan triangle plots represent N, 1e-I, 2e-I and 1.5e-I, respectively. Open and close plots corresponds to D_2A and DA compound, respectively. Purple triangle corresponds to the DA compound exhibits temperature and pressure-induced N-I phase transition. **1**: [{Ru_2(*o*-CF_3PhCO_2)$_4$}(TCNQ)],[1] **2**: [{Ru_2(*o*-CF_3PhCO_2)$_4$}{TCNQ(Me)$_2$}],[1] **3**: [{Ru_2(*o*-CF_3PhCO_2)$_4$}(TCNQF$_4$)],[1] **4**: [{Ru_2(*o*-CH_3PhCO_2)$_4$}(BTDA-TCNQ)],[1] **5**: [{Ru_2(*p*-CH_3PhCO_2)$_4$}(BTDA-TCNQ)],[1] **6**: [{Ru_2(*m*-CH_3PhCO_2)$_4$}(BTDA-TCNQ)],[2,3] **7**: [{$Ru_2(CF_3CO_2)_4$}{TCNQ(MeO)$_2$}],[4] **8**: [{$Ru_2(CF_3CO_2)_4$}{TCNQ(Me)$_2$}],[4] **9**: [{$Ru_2(CF_3CO_2)_4$}(TCNQ)],[4–6] **10**: [{$Ru_2(CF_3CO_2)_4$}(TCNQBr$_2$)],[4,6] **11**: [{$Ru_2(CF_3CO_2)_4$}(TCNQF$_2$)],[4,6] **12**: [{$Ru_2(CF_3CO_2)_4$}(TCNQCl$_2$)],[4,6] **13**: [{Ru_2(2,3,5,6-F_4PhCO_2)$_4$}{DCNQI(Me)$_2$}],[7–9] **14**: [{$Ru_2(CF_3CO_2)_4$}(TCNQF$_4$)],[4,6,10] **15**: [{Ru_2(*o*-$ClPhCO_2$)$_4$}{TCNQ(MeO)$_2$}],[11] **16**: [{Ru_2(*m*-$FPhCO_2$)$_4$}(BTDA-TCNQ)],[3,12,13] **17**: [{Ru_2(*p*-$FPhCO_2$)$_4$}(BTDA-TCNQ)],[14] **18**: [{Ru_2(*o*-$FPhCO_2$)$_4$}(BTDA-TCNQ)],[14] **19**: [{Ru_2(4-Cl-2-$MeOPhCO_2$)$_4$}(BTDA-TCNQ)],[15,16] **20**: [{Ru_2(*o*-$FPhCO_2$)$_4$}{TCNQ(MeO)$_2$}],[17] **21**: [{Ru_2(*m*-$FPhCO_2$)$_4$}{TCNQ(MeO)$_2$}],[17] **22**: [{Ru_2(*p*-$FPhCO_2$)$_4$}{TCNQ(MeO)$_2$}],[17] **23**: [{Ru_2(2,4,6-F_3PhCO_2)$_4$}(TCNQ)],[18] **24**: [{$Ru_2(F_5PhCO_2)_4$}{DCNQI(Me)$_2$}],[8] **25**: [{Ru_2(2,3,5,6-F_4PhCO_2)$_4$}{DCNQI(MeO)$_2$}],[9] **26**: [{Ru_2(2,3,5,6-F_4PhCO_2)$_4$}(BTDA-TCNQ)],[19] **27**: [{$Ru_2(CF_3CO_2)_4$}(BTDA-TCNQ)].[20] **28**: [{Ru_2(3,4-Cl_2PhCO_2)$_4$}{TCNQ(EtO)$_2$}].

Computational Details and redox potential for $TCNQ(EtO)_2$. Theoretical ab initio calculations of $TCNQ(EtO)_2$ was performed using the density functional theory (DFT) formalism, as implemented in the Gaussian 09 software,[21] with the Beck′s three parameter hybrid functional with the correlation functional of Lee, Yang and Parr (B3LYP).[22] 6-31G basis sets with polarization and diffuse functions (6-31+G(d))[23–27] for C, H, N, and O atoms were adopted. The atomic coordinates were optimized. The calculation provided the E_{LUMO}(A) of $TCNQ(EtO)_2$ as −4.5468 eV.

Meanwhile, the first redox potential of $TCNQ(EtO)_2$ were located at −300 mV (vs. Ag/Ag^+ in THF).

Table S1. Crystallographic data for **1-DCE** and **1**.

	**1-DCE**	**1**
T / K	103(1)	103(1)
formula	$C_{46}H_{28}Cl_{10}N_4O_{10}Ru_2$	$C_{44}H_{24}Cl_8N_4O_{10}Ru_2$
formula weight	1353.42	1254.46
crystal system	Triclinic	Triclinic
space group	P−1	P−1
a / Å	9.6890(4)	13.6435(15)
b / Å	11.8638(5)	14.5434(17)
c / Å	12.1018(5)	14.7175(17)
α / deg	68.844(4)	66.425(11)
β / deg	89.177(3)	75.794(10)
γ / deg	81.927(3)	69.636(10)
V / Å^3	1283.47(10)	2490.0(5)
Z	1	2
crystal size / mm^3	0.085×0.057×0.023	0.125×0.088×0.039
D_{calc} / g·cm^{-3}	1.751	1.673
F_{000}	670.00	1240.00
λ / Å	0.71073	0.71073
μ(Mo Kα) / cm^{-1}	11.688	10.941
data measured	9242	17599
data unique	4695	9521
R_{int}	0.0383	0.0500
no. of observations	4695	9521
no. of variables	334	649
$R1$ ($I > 2.00\sigma(I)$)[a]	0.0579	0.0942
R (all reflections)[a]	0.0744	0.1726
$wR2$ (all reflections)[b]	0.1546	0.2713
GOF	1.056	1.023
CCDC No.	1565753	1565754

[a] $R1 = R = \Sigma||F_o| - |F_c||/\Sigma|F_o|$. [b] $wR2 = [\Sigma w(F_o^2 - F_c^2)^2/\Sigma w(F_o^2)^2]^{1/2}$

Table S2. Unit cell parameters and final R_1, wR_2, and good-of-fit (GOF) values for crystallographic analyses taken at several temperatures in the range of 103−270 K.

	T/K	a/Å	b/Å	c/Å	α/°	β/°	γ/°	V/Å^3	R_1 [a]	wR_2 [b]	GOF
**1-DCE**	103	9.6890(4)	11.8638(5)	12.1018(5)	68.844(4)	89.177(3)	81.927(3)	1283.47(10)	0.0579	0.1546	1.056
	151	9.7315(3)	11.8664(4)	12.1111(4)	68.967(3)	89.476(3)	82.126(2)	1291.86(8)	0.0557	0.1504	1.058
	170	9.7596(4)	11.8880(5)	12.1016(6)	68.810(4)	89.690(4)	82.382(3)	1296.19(11)	0.0568	0.1500	1.058
	189	9.7951(4)	11.9004(5)	12.0810(6)	68.664(4)	89.980(4)	82.640(3)	1299.27(11)	0.0554	0.1414	1.069
	199 [b]	9.8036(4)	11.8867(5)	12.0796(6)	68.774(4)	90.047(4)	82.650(3)	1299.69(11)	0.0564	0.1463	1.071
	208 [b]	9.8149(4)	11.8750(5)	12.0838(6)	68.914(4)	90.098(4)	82.604(3)	1301.40(11)	0.0564	0.1447	1.074
	213 [b]	9.8240(3)	11.8697(4)	12.0892(5)	69.000(4)	90.125(3)	82.575(3)	1303.24(9)	0.0562	0.1492	1.065
	218 [b]	9.8359(5)	11.8563(6)	12.0931(7)	69.161(5)	90.160(4)	82.502(4)	1304.90(13)	0.0624	0.1630	1.066
	223 [b]	9.8758(4)	11.7969(6)	12.0958(7)	69.858(5)	90.267(4)	82.070(4)	1308.31(12)	0.0632	0.1673	1.061
	227 [b]	9.9027(4)	11.7670(5)	12.1034(6)	70.189(4)	90.321(4)	81.892(4)	1311.45(11)	0.0598	0.1560	1.062
	232 [b]	9.9228(4)	11.7666(6)	12.1206(7)	70.333(5)	90.350(4)	81.825(4)	1316.86(12)	0.0628	0.1686	1.053
	237 [b]	9.9818(3)	11.6464(4)	12.1339(5)	71.504(3)	90.453(3)	81.112(3)	1319.22(9)	0.0605	0.1652	1.061
	242 [b]	9.9878(4)	11.6386(5)	12.1362(6)	71.540(4)	90.491(4)	81.112(4)	1319.61(11)	0.0645	0.1733	1.061
	246 [b]	9.9966(4)	11.6338(5)	12.1423(7)	71.601(4)	90.513(4)	81.095(4)	1321.27(11)	0.0649	0.1728	1.065
	251 [b]	10.0019(5)	11.6257(5)	12.1444(7)	71.646(5)	90.553(4)	81.092(4)	1321.56(12)	0.0639	0.1736	1.069
	270 [b]	10.0231(4)	11.6006(5)	12.1630(7)	71.838(5)	90.635(4)	81.061(4)	1324.79(12)	0.0617	0.1758	1.052
**1**	103	13.6435(15)	14.5434(17)	14.7175(17)	66.425(11)	75.794(10)	69.636(10)	2490.0(5)	0.0942	0.2713	1.023
	199	13.7066(16)	14.6147(17)	14.7608(17)	66.319(11)	75.691(10)	69.809(11)	2521.1(6)	0.0983	0.2853	1.022
	232	13.7165(17)	14.6378(18)	14.7725(18)	66.255(12)	75.647(11)	69.911(11)	2529.0(6)	0.1002	0.3040	1.025
	242	13.7416(18)	14.6726(19)	14.7949(18)	66.184(12)	75.636(11)	69.954(12)	2542.7(6)	0.1049	0.3153	1.033
	270	13.7668(18)	14.7129(19)	14.8166(19)	66.124(13)	75.561(11)	69.991(12)	2557.1(6)	0.1046	0.3246	1.024

[a] $R_1 = R = \Sigma||F_o| - |F_c||/\Sigma|F_o|$. [b] $wR_2 = [\Sigma w(F_o^2 - F_c^2)^2/\Sigma w(F_o^2)^2]^{1/2}$ [b] For easy comparison, lattice constants in non-standard cell-system was shown here (α, $\gamma < 90°$ while $\beta > 90°$), which can be transformed from the original standard cell (α, β, $\gamma > 90°$) by the following relationships; ***a***’ = −***a***, ***b***’ = ***b***, and ***c***’ = −***c***, where ***a***, ***b*** and ***c*** are the standard axis vectors based on IUCR rule, and ***a***’, ***b***’, and ***c***’ are the transformed ones.

Table S3. Selected bond length (Å), angles, and the estimation of overlap integral at several temperatures in the range of 103−270 K.

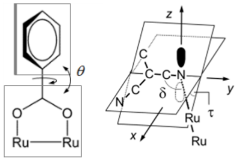

[a] Dihedral angle between two least-square plane formed by (Ru−Ru−N≡C−C) and (C−C−(C≡N)$_2$), [b] dihedral angle between the least squares planes defined by the phenyl ring of benzoate ligand and a carboxylate-bridging mode (atom set of Ru_2O_2C) with benzoic acid Containing C1−C7, [c] benzoic acid Containing C9−C14, [d] calculated by $(\sin\delta\cdot\sin\tau)^2$, [e] calculated by $\{1-(\sin\delta\cdot\sin\tau)^2\}^{0.5}$.

	T/K	Ru−Ru/Å	Ru−O$_{Ave}$/Å	Ru−N/Å	Ru−N−C (δ)/°	τ/° [a]	*θ* set-1/° [b]	*θ* set-2/° [c]	A_σ [d]	A_π [e]
**1-DCE**	103	2.2889(7)	2.020(4)	2.201(6)	157.8(5)	25.07	14.62	9.91	0.03	0.99
	151	2.2882(7)	2.020(4)	2.204(5)	157.4(4)	24.28	14.66	9.26	0.02	0.99
	170	2.2887(7)	2.020(4)	2.206(6)	157.7(5)	24.02	15.01	8.98	0.02	0.99
	189	2.2886(7)	2.019(4)	2.214(6)	157.5(5)	22.98	14.93	8.61	0.02	0.99
	199	2.2878(7)	2.021(4)	2.205(5)	158.2(5)	23.27	15.25	8.64	0.02	0.99
	208	2.2872(7)	2.020(4)	2.209(6)	158.2(5)	22.56	14.94	8.61	0.02	0.99
	213	2.2872(7)	2.020(4)	2.211(5)	157.7(4)	21.85	14.90	8.45	0.02	0.99
	218	2.2867(7)	2.024(4)	2.216(6)	158.3(5)	21.19	14.85	8.03	0.02	0.99
	223	2.2826(8)	2.032(4)	2.231(7)	158.8(5)	18.70	14.20	6.91	0.01	0.99
	227	2.2833(7)	2.038(4)	2.233(6)	159.7(5)	17.17	13.80	6.56	0.01	0.99
	232	2.2841(8)	2.044(4)	2.234(7)	159.6(5)	16.48	13.85	6.38	0.01	1.00
	237	2.2768(7)	2.056(4)	2.267(6)	159.9(5)	12.78	12.61	4.63	0.01	1.00
	242	2.2777(8)	2.054(4)	2.269(7)	160.3(5)	13.09	12.58	4.72	0.01	1.00
	246	2.2770(8)	2.056(4)	2.266(7)	160.4(5)	12.87	12.49	4.39	0.01	1.00
	251	2.2765(8)	2.056(4)	2.280(7)	160.0(6)	12.14	12.42	4.42	0.01	1.00
	270	2.2767(8)	2.056(4)	2.272(6)	160.0(5)	11.68	12.47	4.34	0.00	1.00
**1** (Ru1)	103	2.2770(11)	2.055(9)	2.276(9)	153.8(8)	52.61	10.78	3.21	0.12	0.94
	199	2.2724(12)	2.057(9)	2.304(9)	153.1(9)	51.08	10.74	3.35	0.12	0.94
	232	2.2722(12)	2.056(10)	2.292(10)	155.0(10)	50.27	10.31	3.68	0.11	0.95
	242	2.2739(12)	2.055(10)	2.288(10)	154.8(9)	50.61	10.63	3.07	0.11	0.94
	270	2.2775(12)	2.064(10)	2.297(10)	156.1(10)	48.99	10.47	2.93	0.09	0.95
**1** (Ru2)	103	2.2498(15)	2.063(11)	2.162(15)	152(2)	33.39	9.51	4.77	0.07	0.97
	199	2.2532(15)	2.060(12)	2.146(17)	153(2)	33.21	9.92	4.70	0.06	0.97
	232	2.2505(17)	2.064(13)	2.163(18)	153(2)	32.29	10.11	5.15	0.06	0.97
	242	2.2504(17)	2.067(12)	2.149(18)	153(2)	32.25	9.25	5.04	0.06	0.97
	270	2.2514(17)	2.071(13)	2.173(19)	152(3)	32.73	9.68	5.00	0.06	0.97

Table S4. Bond distances (Å) in $TCNQ(EtO)_2$ subunit at several temperatures in the range of 103−270 K.

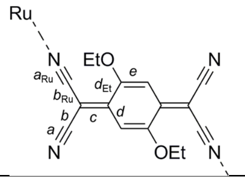

	T/K	a_{Ru}	a	a_{ave}	b_{Ru}	b	b_{ave}	c	d_{Et}	d	d_{ave}	e	$c/(b_{ave}+d_{ave})$	ρ^a
**I**		1.140(1)			1.441(1)			1.374(3)	1.448(4)			1.346(3)	0.476	0
**II**		1.153(7)			1.416(8)			1.420(1)	1.423(3)			1.373(1)	0.576	−1
**1-DCE**	103	1.152(9)	1.152(9)	1.152(9)	1.416(10)	1.411(9)	1.414(10)	1.432(10)	1.428(7)	1.406(9)	1.417(8)	1.359(10)	0.506	−1.247
	151	1.153(9)	1.153(9)	1.153(9)	1.411(9)	1.420(8)	1.416(8)	1.421(9)	1.427(7)	1.411(9)	1.419(8)	1.363(9)	0.501	−1.056
	170	1.143(9)	1.154(9)	1.148(9)	1.422(10)	1.407(9)	1.414(10)	1.428(10)	1.430(7)	1.412(9)	1.421(8)	1.353(9)	0.504	−1.151
	189	1.147(9)	1.152(9)	1.150(9)	1.415(9)	1.412(9)	1.414(9)	1.420(10)	1.430(7)	1.416(9)	1.423(8)	1.351(9)	0.501	−1.026
	199	1.135(9)	1.146(10)	1.140(10)	1.428(10)	1.416(9)	1.422(10)	1.416(10)	1.429(7)	1.406(9)	1.418(9)	1.353(9)	0.499	−0.946
	208	1.142(9)	1.146(10)	1.144(10)	1.414(10)	1.431(9)	1.422(10)	1.421(10)	1.419(7)	1.405(9)	1.412(8)	1.354(10)	0.501	−1.056
	213	1.146(9)	1.156(10)	1.151(10)	1.413(10)	1.417(9)	1.415(10)	1.428(10)	1.429(7)	1.410(9)	1.420(8)	1.351(9)	0.504	−1.158
	218	1.134(10)	1.145(11)	1.140(10)	1.423(11)	1.415(10)	1.419(10)	1.426(11)	1.435(8)	1.413(10)	1.424(9)	1.345(10)	0.502	−1.066
	223	1.137(11)	1.144(12)	1.140(12)	1.421(11)	1.418(10)	1.420(10)	1.416(11)	1.433(9)	1.416(10)	1.424(10)	1.356(11)	0.498	−0.913
	227	1.137(10)	1.146(11)	1.142(10)	1.430(11)	1.425(10)	1.428(10)	1.401(11)	1.436(8)	1.421(10)	1.428(9)	1.356(10)	0.490	−0.607
	232	1.132(11)	1.137(12)	1.134(12)	1.444(12)	1.417(11)	1.430(12)	1.413(11)	1.437(8)	1.421(10)	1.429(9)	1.356(11)	0.494	−0.756
	237	1.141(10)	1.141(11)	1.141(10)	1.443(10)	1.426(9)	1.434(10)	1.393(10)	1.445(8)	1.429(10)	1.437(9)	1.340(10)	0.485	−0.380
	242	1.136(11)	1.139(12)	1.138(12)	1.443(11)	1.419(10)	1.431(10)	1.389(11)	1.441(9)	1.429(11)	1.435(10)	1.353(11)	0.485	−0.361
	246	1.133(11)	1.145(12)	1.139(12)	1.443(11)	1.429(10)	1.436(10)	1.386(11)	1.446(9)	1.428(11)	1.437(10)	1.342(11)	0.482	−0.268
	251	1.122(11)	1.135(12)	1.128(12)	1.445(12)	1.425(11)	1.435812)	1.397(12)	1.443(9)	1.425(11)	1.434(10)	1.340(11)	0.487	−0.456
	270	1.128(10)	1.141(11)	1.134(10)	1.443(11)	1.420(10)	1.432(10)	1.392(11)	1.452(8)	1.426(10)	1.439(9)	1.330(10)	0.485	−0.373
**1**^{b}	103	1.134(14)	1.15(2)	1.14(2)	1.418(16)	1.45(2)	1.43(2)	1.360(15)	1.45(2)	1.439(15)	1.44(2)	1.356(15)	0.472	0.147
	199	1.140(15)	1.14(3)	1.14(2)	1.410(17)	1.44(3)	1.42(2)	1.361(15)	1.45(2)	1.439(15)	1.44(2)	1.351(15)	0.474	0.070
	232	1.122(16)	1.15(3)	1.14(2)	1.413(17)	1.43(3)	1.42(2)	1.372(16)	1.45(2)	1.433(16)	1.44(2)	1.355(16)	0.479	−0.135
	242	1.147(16)	1.14(3)	1.14(2)	1.398(17)	1.46(3)	1.43(2)	1.359(16)	1.45(2)	1.430(16)	1.44(2)	1.360(16)	0.474	0.096
	270	1.116(17)	1.14(3)	1.13(2)	1.415(19)	1.44(3)	1.43(2)	1.356(18)	1.47(2)	1.432(17)	1.45(2)	1.342(17)	0.471	0.205
**1**^{c}	103	1.18(2)	1.15(2)	1.16(2)	1.44(2)	1.40(3)	1.42(2)	1.377(18)	1.45(2)	1.42(2)	1.44(2)	1.373(17)	0.482	−0.263
	199	1.15(2)	1.16(3)	1.16(2)	1.47(3)	1.41(3)	1.44(3)	1.368(19)	1.45(3)	1.41(2)	1.43(2)	1.398(18)	0.477	−0.028
	232	1.17(3)	1.15(3)	1.16(3)	1.46(3)	1.39(3)	1.42(3)	1.37(2)	1.42(3)	1.41(3)	1.42(3)	1.394(19)	0.482	−0.267
	242	1.17(3)	1.16(3)	1.16(3)	1.47(3)	1.39(3)	1.43(3)	1.36(2)	1.44(3)	1.42(3)	1.43(3)	1.391(19)	0.476	0.019
	270	1.15(3)	1.17(3)	1.16(3)	1.48(3)	1.40(3)	1.44(3)	1.36(2)	1.43(3)	1.40(3)	1.42(3)	1.40(2)	0.476	−0.015

I: TCNQ,[28] **II**: RbTCNQ.[29] $^a\rho = A_H[c/(b + d)] + B_H$ with $A_H = -41.667$ and $B_H = 19.833$, ^{b} TCNQ unit in the chain containing Ru(1), ^{c} TCNQ unit in the chain containing Ru(2).

Table S5. Relevant geometrical parameters for **1-DCE** related to chain packing.

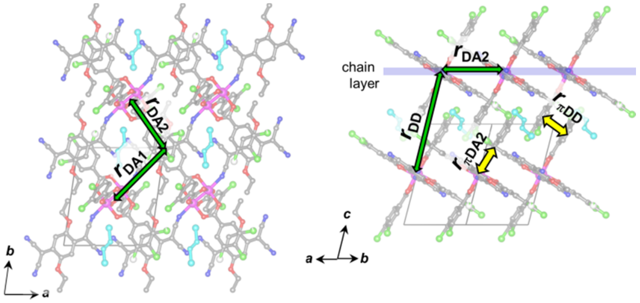

	T/K	Intra-chain distance between the center of $[Ru_2]$ and TCNQ(EtO)$_2$ (r_{DA1} = {$\|\boldsymbol{a} + \boldsymbol{b}\|/2$}) / Å	Inter-chain distance between the center of $[Ru_2]$ and TCNQ(EtO)$_2$ along [1−10] (r_{DA2} = {$\|\boldsymbol{a} - \boldsymbol{b}\|/2$}) / Å	π stack distance along [1−10][a] ($r_{\pi DA2}$) / Å	Inter-chain distance along [001] (r_{DD} = $\|\boldsymbol{c}\|$) / Å	π stack distance along [001] [b] ($r_{\pi DD}$) / Å	Void volume / Å^3
**1-DCE**	103	8.169	7.112	3.519	12.1018	3.459	118.2 (9.2%)
	151	8.172	7.139	3.534	12.1111	3.475	123.1 (9.5%)
	170	8.175	7.173	3.541	12.1016	3.492	124.2 (9.6%)
	189	8.177	7.206	3.553	12.0810	3.500	127.2 (9.8%)
	199	8.173	7.204	3.558	12.0796	3.501	127.2 (9.8%)
	208	8.175	7.200	3.551	12.0838	3.506	129.1 (9.9%)
	213	8.178	7.198	3.557	12.0892	3.509	129.8 (10.0%)
	218	8.182	7.192	3.558	12.0931	3.504	130.1 (10.0%)
	223	8.198	7.151	3.560	12.0958	3.496	132.6 (10.1%)
	227	8.207	7.135	3.559	12.1034	3.493	136.0 (10.4%)
	232	8.218	7.136	3.566	12.1206	3.495	138.5 (10.5%)
	237	8.234	7.060	3.546	12.1339	3.471	143.9 (10.9%)
	242	8.233	7.059	3.549	12.1362	3.482	145.1 (11.0%)
	246	8.235	7.058	3.550	12.1423	3.473	146.0 (11.0%)
	251	8.234	7.057	3.551	12.1444	3.465	145.7 (11.0%)
	270	8.234	7.052	3.560	12.1630	3.487	150.0 (11.3%)

[a] Defined by the distance between TCNQ plane and the centroid of phenyl ring of the benzoate moiety in the neighboring chain, [b] defined by the distance between pi planes of phenyl rings, [c] defined by the distance between the centroids of phenyl rings.

Table S5 (Continued). Relevant geometrical parameters for **1** related to chain packing.

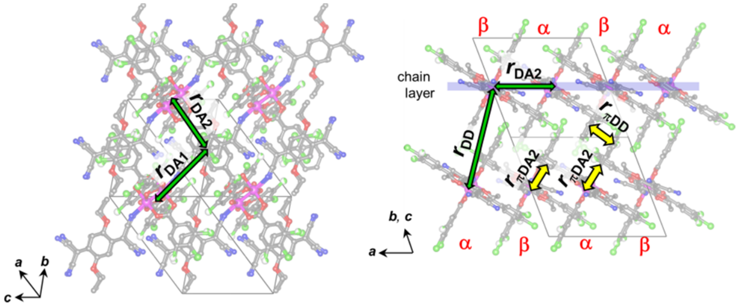

	T/K	Intra-chain distance between the center of [Ru_2] and TCNQ(EtO)$_2$ (r_{DA1} = {|$\boldsymbol{b} - \boldsymbol{c}$|/2}) / Å	Inter-chain distance between the center of [Ru_2] and TCNQ(EtO)$_2$ along [100] (r_{DA2} = |$\boldsymbol{a}$|/2) / Å	π stack distance along [100][a] ($r_{\pi DA2}$) / Å	Inter-chain distance between the center of [Ru_2] and TCNQ(EtO)$_2$ along [1−1−1] (r_{DD} = {|$\boldsymbol{a} - \boldsymbol{b} - \boldsymbol{c}$|/2}) / Å	π stack distance along [1−1−1] [b] ($r_{\pi DD}$) / Å	Void volume / Å^3
**1**	103	8.014	6.822	3.441/3.455	11.713	3.506	159.2 (6.4%)
	199	8.034	6.853	3.472/3.491	11.772	3.540	159.4 (6.3%)
	232	8.037	6.858	3.480/3.485	11.794	3.548	164.1 (6.5%)
	242	8.045	6.871	3.493/3.505	11.824	3.570	164.0 (6.4%)
	270	8.055	6.883	3.514/3.510	11.850	3.575	166.1 (6.5%)

[a] Defined by the distance between TCNQ plane and the centroid of phenyl ring of the benzoate moiety in the neighboring chain. Two values are corresponding to the distance between TCNQ in α-chain and phenyl group in β-chain, and TCNQ in β-chain and phenyl ring in α-chain, respectively, [b] defined by the distance between the centroids of phenyl rings.

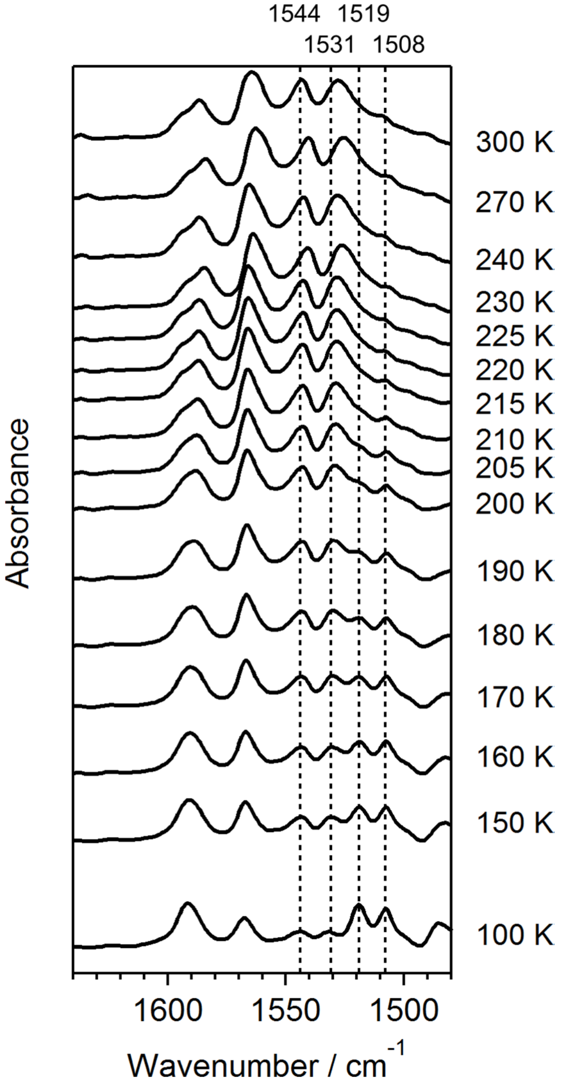

Figure S2. Infrared absorption spectra of **1-DCE** in the wavenumber range of 1480−1640 cm^{-1} measured on nujol mull, where the bands at 1544 and 1531 cm^{-1} correspond to ν(C=C) of the neutral $TCNQ(EtO)_2$ moieties, and the bands 1519 and 1508 cm^{-1} correspond to ν(C=C) of the ionic $TCNQ(EtO)_2$ moieties.

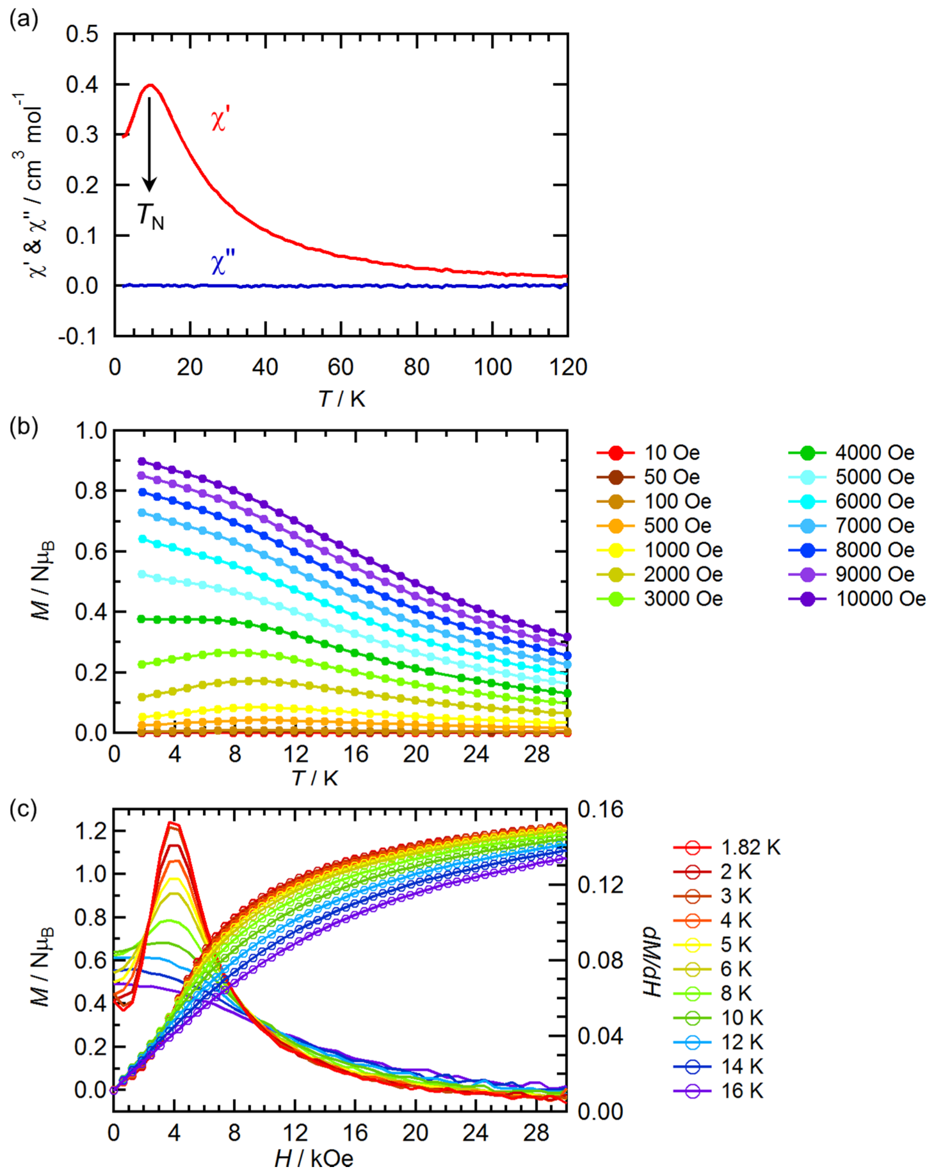

Figure S3. Magnetic properties of **1-DCE** in the low-temperature region. (a) Alternating current (ac) susceptibilities measured at 1 Hz under zero dc field and 3 Oe oscillating field. (b) Field-cooled magnetization (FCM) plots measured at a range of fields. (c) Field dependence of the magnetization ($M{-}H$ plots) and dM/dH plots measured at a range of temperatures.

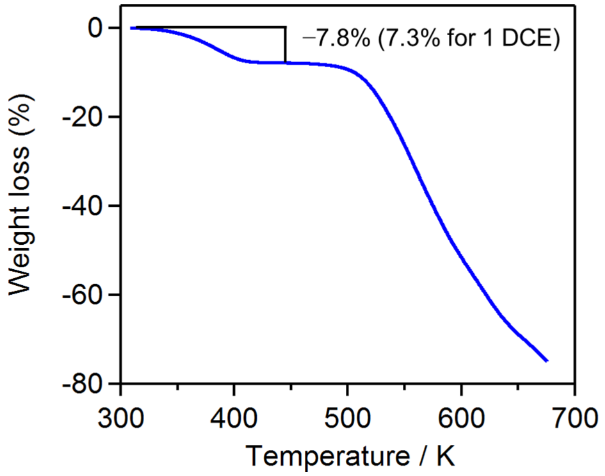

Figure S4. Thermogravimetric analysis (TGA) profiles of **1-DCE** with a heating rate of 5 K min^{-1} under N_2 atmosphere.

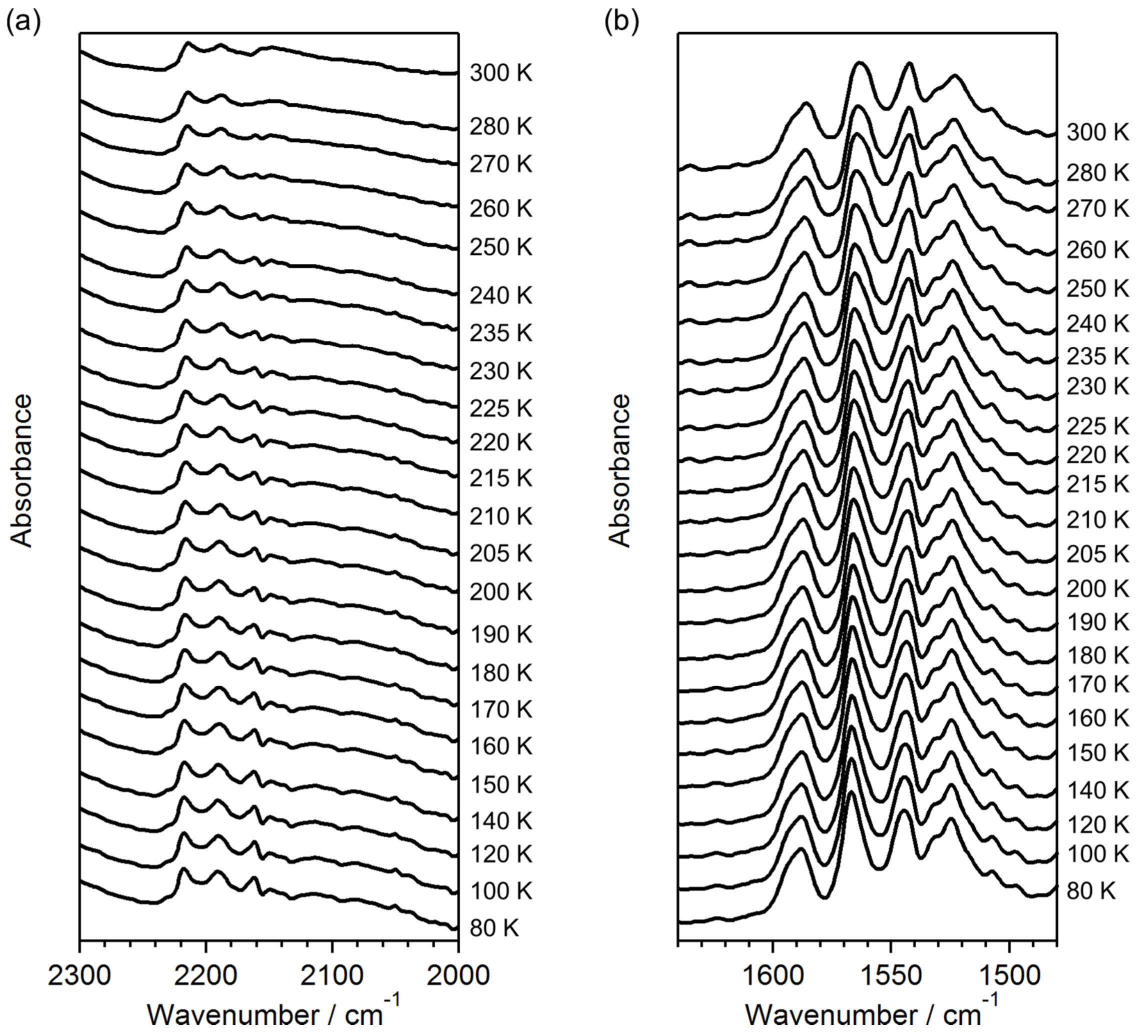

Figure S5. Infrared absorption spectra of **1** in the wavenumber range of 2000−2300 cm^{-1} (a) and 1480−1640 cm^{-1} (b) measured on nujol mull.

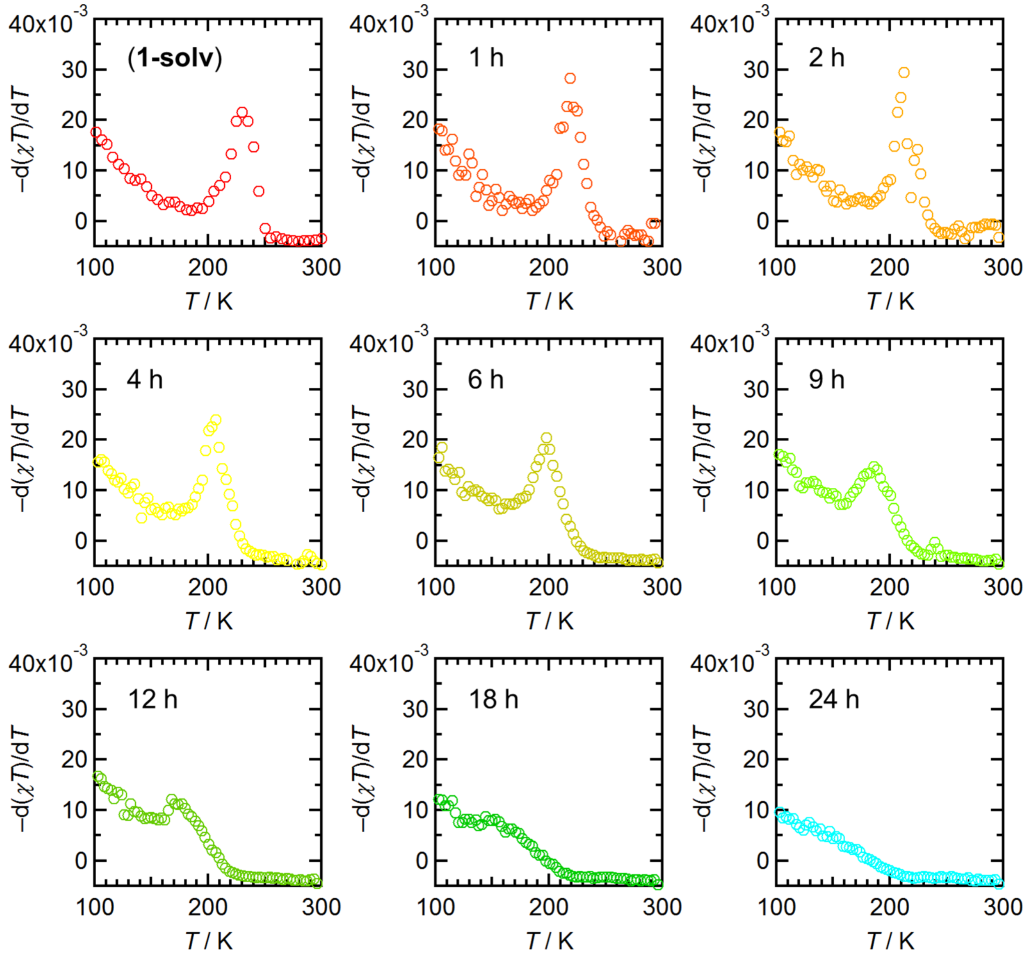

Figure S6. Time course magnetic variation dχT/dT –T plots measured at 1 kOe for **1-DCE**.

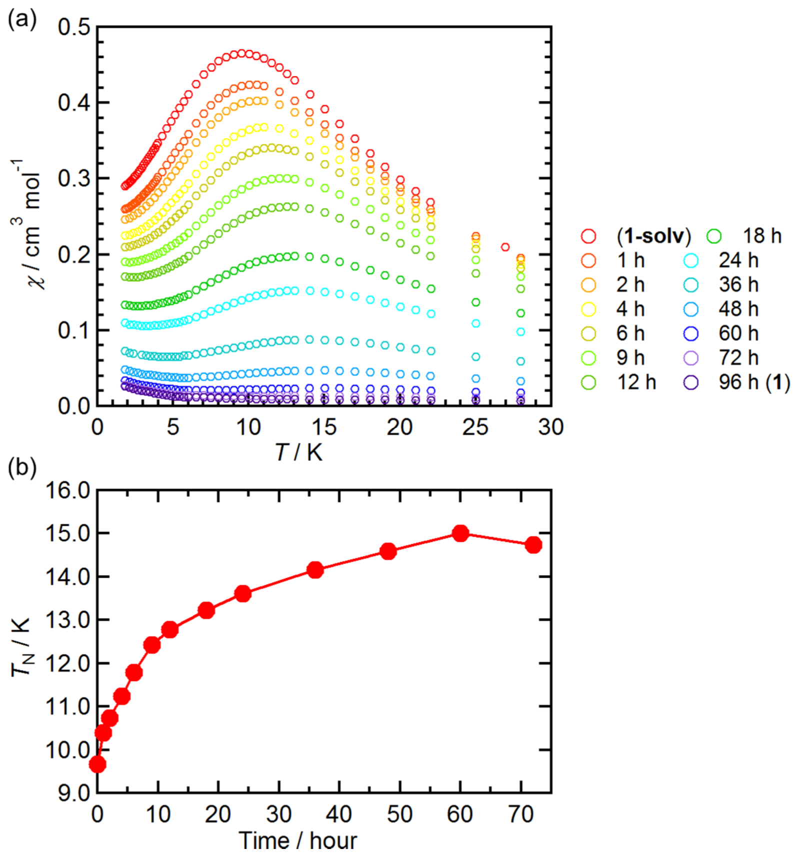

Figure S7. Time course magnetic variation of **1-DCE** in the low-temperature region. (a) Field-cooled magnetization (FCM) plots measured at 1 kOe. (b) time-dependency of the Néel temperature (T_N) defined by the temperature where peak appears in the χ−T plots.

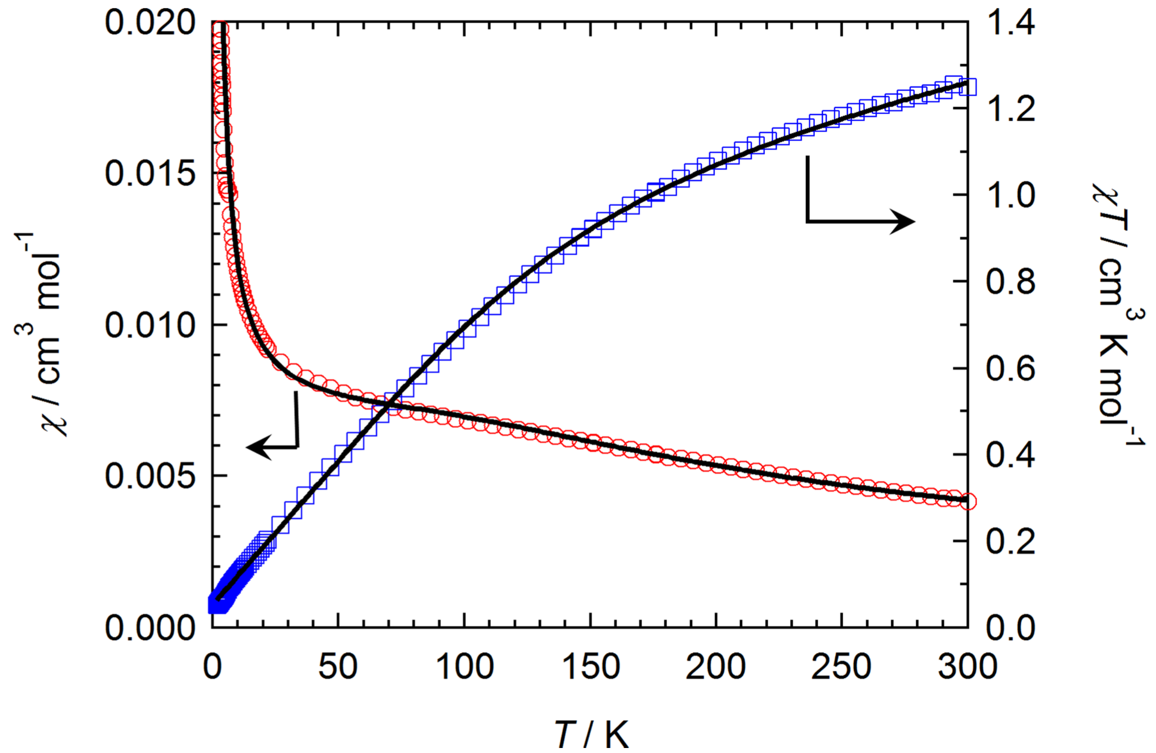

Figure S8. Temperature dependence of magnetic susceptibility χ and the χT producet of **1**, where the solid lines were fitted using a Curie equation with S = 1 taking into account the zero-field splitting (D), the temperature-independent paramagnetism (χ_{TIP}), and impurity (ρ_{imp}) as S = 3/2 with a parameter set of g = 2.0 (fix), D/k_{B} = 348 K, and $\chi_{\mathrm{TIP}} = 1.04(2) \times 10^{-3}$ cm^3 mol^{-1}, and $\rho_{\mathrm{imp}} = 2.88(3) \times 10^{-2}$.

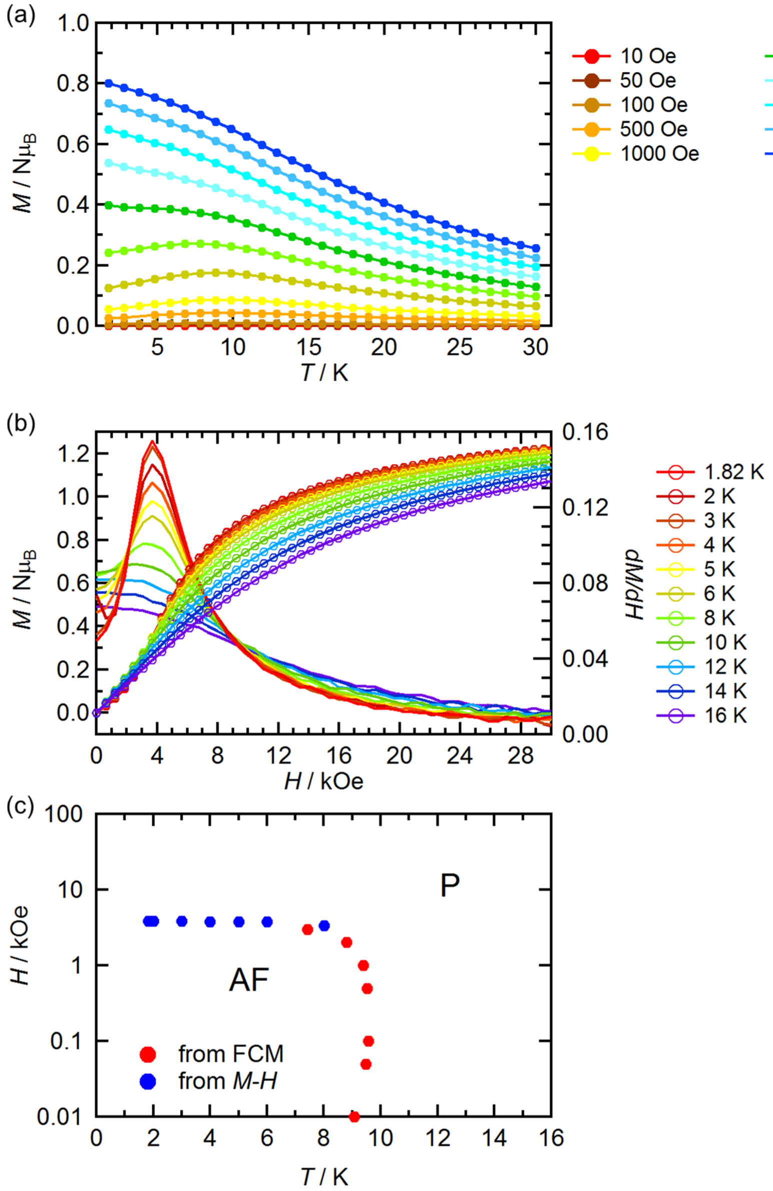

Figure S9. Magnetic properties of **1-DCE'** in the low-temperature region. (a) Field-cooled magnetization (FCM) plots measured at a range of fields. (b) Field dependence of the magnetization (*M*−*H* plots) and d*M*/d*H* plots measured at a range of temperatures. (cs) *H*−*T* phase diagrams.

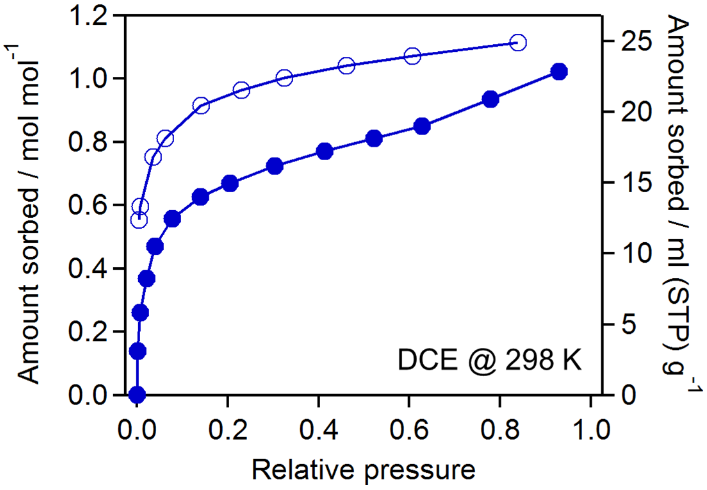

Figure S10. Adsorption (closed) and desorption (open) isotherms of DCE vapor for **1** at 298 K. The lines are guide to eyes.

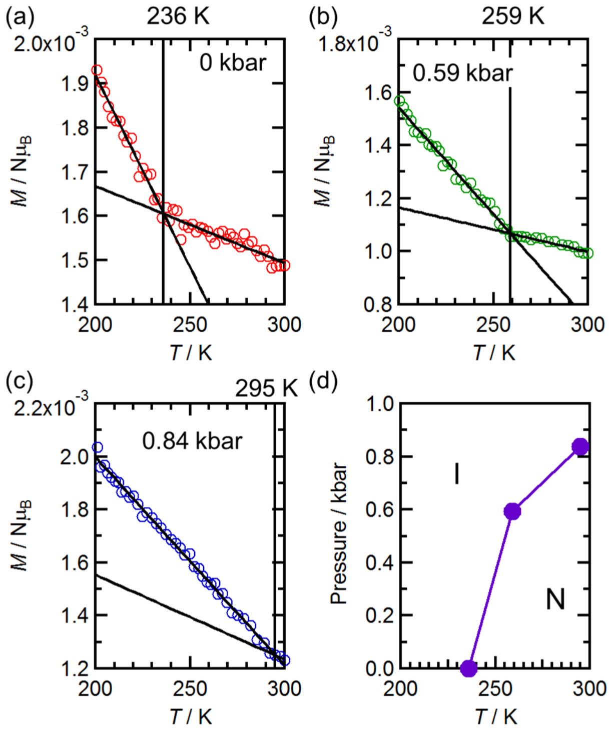

Figure S11. Variations of the magnetization (*M*) of **1-DCE** as a function of temperature measured at several pressures; (a) ambient pressure, (b) 0.59 kbar, and (c) 0.84 kbar. (d) Applied pressure-temperature phase diagrams for **1-DCE**.

References for SI

[1] W. Kosaka, T. Morita, T. Yokoyama, J. Zhang, H. Miyasaka, *Inorg. Chem.* **2015**, *54*, 1518.

[2] H. Miyasaka, T. Morita, M. Yamashita, *Chem. Commun.* **2011**, *47*, 271.

[3] H. Fukunaga, W. Kosaka, H. Miyasaka, *Chem. Lett.* **2014**, *43*, 541.

[4] K. Nakabayashi, M. Nishio, K. Kubo, W. Kosaka, H. Miyasaka, *Dalton Trans.* **2012**, *41*, 6072.

[5] H. Miyasaka, C. S. Campos-Fernández, R. Clérac, K. R. Dunbar, *Angew. Chem. Int. Ed.* **2000**, *39*, 3831.

[6] H. Miyasaka, N. Motokawa, S. Matsunaga, M. Yamashita, K. Sugimoto, T. Mori, N. Toyota, K. R. Dunbar, *J. Am. Chem. Soc.* **2010**, *132*, 1532.

[7] H. Miyasaka, N. Motokawa, T. Chiyo, M. Takemura, M. Yamashita, H. Sagayama, T. Arima, *J. Am. Chem. Soc.* **2011**, *133*, 5338.

[8] K. Nakabayashi, H. Miyasaka, *Chem. Eur. J.* **2014**, *20*, 5121.

[9] K. Nakabayashi, M. Nishio, H. Miyasaka, *Inorg. Chem.* **2016**, *55*, 2473.

[10] H. Miyasaka, T. Izawa, N. Takahashi, M. Yamashita, K. R. Dunbar, *J. Am. Chem. Soc.* **2006**, *128*, 11358.

[11] N. Motokawa, S. Matsunaga, S. Takaishi, H. Miyasaka, M. Yamashita, K. R. Dunbar, *J. Am. Chem. Soc.* **2010**, *132*, 11943.

[12] N. Motokawa, H. Miyasaka, M. Yamashita, K. R. Dunbar, *Angew. Chem. Int. Ed.* **2008**, *47*, 7760.

[13] N. Motokawa, H. Miyasaka, M. Yamashita, *Dalton Trans.* **2010**, *39*, 4724.

[14] N. Motokawa, T. Oyama, S. Matsunaga, H. Miyasaka, M. Yamashita, K. R. Dunbar, *CrystEngComm,* **2009**, *11*, 2121.

[15] M. Nishio, N. Hoshino, W. Kosaka, T. Akutagawa, H. Miyasaka, *J. Am. Chem. Soc.* **2013**, *135*, 17715.

[16] M. Nishio, H. Miyasaka, *Inorg. Chem.* **2014**, *53*, 4716.

[17] H. Fukunaga, T. Yoshino, H. Sagayama, J. Yamaura, T. Arima, W. Kosaka, H. Miyasaka, *Chem. Commun.* **2015**, *51*, 7795.

[18] W. Kosaka, H. Fukunaga, H. Miyasaka, *Inorg. Chem.* **2015**, *54*, 10001.

[19] K. Taniguchi, K. Narushima, J. Mahin, W. Kosaka, H. Miyasaka, *Angew. Chem. Int. Ed.* **2016**, *55*, 5238.

[20] K. Taniguchi, K. Narushima, H. Sagayama, W. Kosaka, N. Shito, H. Miyasaka, *Adv. Func. Mater.* **2017**, *27*, 1604990.

[21] Gaussian 09, Revision B.01, M. J. Frisch, G. W. Trucks, H. B. Schlegel, G. E. Scuseria, M. A. Robb, J. R. Cheeseman, G. Scalmani, V. Barone, B. Mennucci, G. A. Petersson, H. Nakatsuji, M. Caricato, Li, X.; H. P. Hratchian, A. F. Izmaylov, J. Bloino, G. Zheng, J. L.

Sonnenberg, M. Hada, M. Ehara, K. Toyota, R. Fukuda, J. Hasegawa, M. Ishida, T. Nakajima, Y. Honda, O. Kitao, H. Nakai, T. Vreven, J. A. Montgomery, Jr., J. E. Peralta, F. Ogliaro, M. Bearpark, J. J. Heyd, E. Brothers, K. N. Kudin, V. N. Staroverov, R. Kobayashi, J. Normand, K. Raghavachari, A. Rendell, J. C. Burant, S. S. Iyengar, J. Tomasi, M. Cossi, N. Rega, J. M. Millam, M. Klene, J. E. Knox, J. B. Cross, V. Bakken, C. Adamo, J. Jaramillo, R. Gomperts, R. E. Stratmann, O. Yazyev, A. J. Austin, R. Cammi, C. Pomelli, J. W. Ochterski, R. L. Martin, K. Morokuma, V. G. Zakrzewski, G. A. Voth, P. Salvador, J. J. Dannenberg, S. Dapprich, A. D. Daniels, Ö. Farkas, J. B. Foresman, J. V. Ortiz, J. Cioslowski and D. J. Fox, Gaussian, Inc., Wallingford CT, **2009**.

[22] A. D. Becke, *J. Chem. Phys.* **1993**, *98*, 5648.

[23] P. C. Hariharan, J. A. Pople, *Theoret. Chimica Acta,* **1973**, *28*, 213.

[24] M. M. Francl, W. J. Petro, W. J. Hehre, J. S. Binkley, M. S. Gordon, D. J. DeFrees, J. A. Pople, *J. Chem. Phys.* **1982**, *77*, 3654.

[25] T. Clark, J. Chandrasekhar, P. V. R. Schleyer, *J. Comp. Chem.* **1983**, *4*, 294.

[26] R. Krishnam, J. S. Binkley, R. Seeger, J. A. Pople, *J. Chem. Phys.* **1980**, *72*, 650.

[27] P. M. W. Gill, B. G. Johnson, J. A. Pople, M. J. Frisch, *Chem. Phys. Lett.* **1992**, *197*, 499.

[28] R. E. Long, R. A. Sparks, K. N. Trueblood, *Acta Cryst.* **1965**, *18*, 932.

[29] C. J. Fritche, Jr., P. Arthur, Jr., *Acta Cryst.* **1966**, *21*, 139.
